# Korean dragon pseudoscorpions revisited: a taxonomic review of Allochthonius (Pseudoscorpiones: Pseudotyrannochthoniidae) Chamberlin, 1929 with three new species

**DOI:** 10.7717/peerj.21332

**Published:** 2026-06-02

**Authors:** Kyung-Hoon Jeong, Danilo Harms, Sora Kim

**Affiliations:** 1 Department of Biology, Faculty of Mathematics, Informatics and Natural Sciences, University of Hamburg, Hamburg, Hamburg, Germany; 2 Museum of Nature Hamburg–Zoology, Leibniz Institute for the Analysis of Biodiversity Change, Hamburg, Hamburg, Germany; 3 Harry Butler Institute, Murdoch University, Murdoch, Western Australia, Australia; 4 Department of Zoology and Entomology, University of Free State, Bloemfontein, South Africa; 5 Lab. of Insect Phylogenetics & Evolution, Department of Bioenvironmental Chemistry, Jeonbuk National University, Jeonju, Jeonju, Republic of South Korea; 6 Department of Agricultural Convergence Technology, Jeonbuk National University, Jeonju, Jeonju, Republic of South Korea

**Keywords:** DNA barcoding, Republic of Korea, Pseudoscorpiones, Subspecies, Systematics, Taxonomy

## Abstract

*Allochthonius* (Pseudoscorpiones: Pseudotyrannochthoniidae) is a genus widely distributed throughout East Asia. In Korea, only three species in this genus have been recorded to date: *Allochthonius buanensis* Lee, 1982; *Allochthonius coreanus* Morikawa, 1970; and *Allochthonius opticus* Ellingsen, 1907. Here, we review the Korean fauna of *Allochthonius* and describe three new species for the country: *Allochthonius jungsuni* **sp. nov.**, *Allochthonius maximus* **sp. nov.**, and *Allochthonius rufimanus* **sp. nov**. We also exclude *Allochthonius opticus* –a species originally described from Japan—from the country record and redescribe two Korean recorded species, *Allochthonius buanensis* and *Allochthonius coreanus*. Following up on our previous taxonomic work that elevated several subspecies of Japanese pseudotyrannochthoniids originally described by Kuniyasu Morikawa to species level, we elevate eight former subspecies to full species rank: *A. ishikawai deciclavatus* Morikawa, 1956 = *A. deciclavatus* Morikawa, 1956, **stat. nov.**, *A. ishikawai ishikawai* Morikawa, 1954 = *A. ishikawai* Morikawa, 1954, *A. ishikawai kyushuensis* Morikawa, 1960 = *A. kyushuensis* Morikawa, 1960, **stat. nov.**, *A. ishikawai shiragatakiensis* Morikawa, 1954 = *A. shiragatakiensis* Morikawa, 1954, **stat. nov.**, *A. ishikawai uenoi* Morikawa, 1956 = *A. uenoi* Morikawa, 1956, **stat. nov.**, and *A. ishikawai uyamadensis* Morikawa, 1954 = *A. uyamadensis* Morikawa, 1954, **stat. nov.**; *A. opticus opticus* Ellingsen, 1907 = *A. opticus* Ellingsen, 1907, and *A. opticus troglophilus* Morikawa, 1956 = *A. troglophilus* Morikawa, 1956, **stat. nov.**. This elevates the total number of *Allochthonius* species from 40 to 43, of which five species are presently known from the Korean Peninsula.

## Introduction

The pseudoscorpion family Pseudotyrannochthoniidae Beier, 1932 represents one of the most basal lineages among the 26 recognized families of Pseudoscorpiones (Benavides et al., 2019; World Pseudoscorpiones Catalog, 2022). Members of this family are commonly referred to as “dragon pseudoscorpions” due to their markedly enlarged chelicerae (Harms et al., 2024). Pseudotyrannochthoniidae is diagnosed by the presence of trichobothria *ib* and *isb* positioned at the base of the fixed chelal finger, in combination with coxal spines restricted to coxa I (Harms & Harvey, 2013; Hou & Zhang, 2024). Pseudotyrannochthoniidae currently comprises seven genera and 108 species: *Afrochthonius* Beier, 1930; *Allochthonius* Chamberlin, 1929; *Centrochthonius* Beier, 1931; *Karrichthonius* Harms et al., 2025; *Pseudotyrannochthonius* Beier, 1930; *Selachochthonius* Chamberlin, 1929; and *Spelaeochthonius* Morikawa, 1954; World Pseudoscorpiones Catalog, 2022; Lin et al., 2025). Three of these genera—*Allochthonius* (China, Japan, Russia, the Republic of Korea, Taiwan), *Centrochthonius* (China, Kyrgyzstan, Nepal), and *Spelaeochthonius* (China, Japan, the Republic of Korea)—are distributed across Asia. Species within these genera typically exhibit small distribution ranges (Harms et al., 2024; Hou & Zhang, 2024) and are most frequently encountered in the leaf litter of mesic forest ecosystems, although numerous species are troglobitic and occur exclusively in cave habitats (*e.g.*, You et al., 2022; Jeong et al., 2025).

Dated molecular phylogenetic data indicates that the genus *Allochthonius* (Chamberlin, 1929) originated in Asia during the Early Cretaceous (Harms et al., 2024). There is one fossil species dating back to the Eocene of Europe (33.7–33.9 Ma) (Schwarze et al., 2021), but all 40 extant species are distributed in eastern Asia: 15 species in China, 14 in Japan, seven in Taiwan, three in the Republic of Korea, and one in Russia (World Pseudoscorpiones Catalog, 2022; Lin et al., 2025). Species of *Allochthonius* can be diagnosed based on variable numbers of carapacal setae (18–28; Sakayori, 2000; Viana & Ferreira, 2021; You et al., 2022) and the presence of “fan-shaped” or “spray-shaped” coxal spines (Chamberlin, 1929; Morikawa, 1960; Morikawa, 1962; Harvey & Harms, 2022; Hou & Zhang, 2024). To date, three species of *Allochthonius* have been formally recorded in the Republic of Korea: the “widespread” morphospecies *Allochthonius buanensis* Lee, 1982 (Type locality: Buan, Jeollabuk-do), *Allochthonius coreanus* (Type locality: Sinryeong-gul Cave in Gangwon-do), and *Allochthonius opticus*; a species originally described from Okayama, Japan but also recorded from Korea by Lee & Seo, 1995 (Morikawa, 1970; Lee, 1982; Lee & Seo, 1995). However, a recent barcoding study has indicated that the diversity of *Allochthonius* in Korea is clearly much higher and some of the most common species (*e.g.*, *Allochthonius buanensis* Lee, 1982) clearly represent species complexes in molecular phylogenies or DNA barcoding studies (Ohira et al., 2018).

Despite being the most commonly collected pseudoscorpion genus in the Republic of Korea (Lee & Seo, 1995; Hong, 1996), the diversity of *Allochthonius* in this region has not been critically reassessed since the 1990s. All previous Korean records were based on limited morphological examinations, and the type materials used in these studies have since been lost (Y. Hong, 2023, pers. comm.), rendering direct comparisons with newly collected material impossible. Recent molecular and taxonomic studies on pseudotyrannochthoniids across East Asia have consistently demonstrated that species in this family exhibit narrow-range endemism and low dispersal capacity, and that broad morphospecies concepts often mask considerable cryptic diversity (Ohira et al., 2018; Harms et al., 2024; Harms et al., 2025; Lin et al., 2025). We will test the hypothesis that the species richness of *Allochthonius* on the Korean Peninsula has been substantially underestimated due to cryptic speciation and narrow-range endemism. To address this, we employ an integrative taxonomic framework combining detailed morphological examination using stereomicroscopy and scanning electron microscopy (SEM) with COI DNA barcoding data, thereby providing two independent lines of evidence for species delimitation.

Herein, we review the Korean fauna of *Allochthonius* and describe three new epigean species from leaf litter habitats. We also correct a previous misidentification and remove *Allochthonius opticus* from the Korean record and re-describe both *A. buanensis* and *A. coreanus*. Following up on previous work that elevated several subspecies of Japanese pseudotyrannochthoniids to full species rank (You et al., 2022), we complete this work and elevate eight previously recognized subspecies of *Allochthonius* to full species rank, this updating species concepts in light of recent taxonomic and molecular work on this family.

## Materials and methods

### Morphological identification

The specimens used for this study are deposited in the following institutions: Museum of Nature Hamburg, Hamburg, Germany (ZMH), National Institute for Biological Resources, Incheon, Republic of Korea (NIBR), and Jeonbuk National University, Jeonju, Republic of Korea (JBNU). All specimens are stored in 100% ethanol.

All specimens in this study were examined using a Leica Z16 APO stereomicroscope. Habitus images were captured using a Leica Z16 APO stereomicroscope and Dhyana 400 DC (4M) sCMOS camera (TUCSEN, Fuzhou, China) and using Mosaic Analysis Software 2.4. Images of appendages were captured using a Leica M205A stereomicroscope and a Leica DMC4500 digital camera, using Leica Application Suite X (LASX) ver. 3.0.1. Appendages were drawn using Adobe Illustrator 2026 (Adobe Inc.). Scanning electron micrographs (SEM) were pictured using a Hitachi TM4000Plus scanning electron micrograph system.

Measurements and terminology follow the following studies (Chamberlin, 1931; Harvey, 1991; Judson, 2007; Kolesnikov, Przhiboro & Turbanov, 2023). Abbreviations for chelal trichobothria: ***b***–basal, ***sb***–subbasal, ***st***–subterminal, ***t***–terminal, ***ib***–internal basal, ***isb***–internal subbasal, ***eb***–external basal, ***esb***–external subbasal, ***it***–internal terminal, ***ist*** –internal subterminal, ***et***–external terminal, ***est***–external subterminal, ***xs***–duplex trichobothria. Abbreviations for carapacal setae: ***A1***–1st anterior seta, ***A2***–2nd anterior seta, ***A3***–3rd anterior seta, ***A4***–4th anterior seta, ***A5***–5th anterior seta, ***Ol***–lateral ocular seta, ***Om1***–1st medial ocular seta, ***Om2***–2nd medial ocular seta, ***Ml***–medialateral seta, ***Mm1***–1st medial seta, ***Mm2***–2nd medial seta, ***Il***–intermedialateral seta, ***Pl***–posterolateral seta, ***Pm***–posteromedial seta.

### Molecular analyses

To generate an integrated taxonomic hypothesis, all five Korean species of *Allochthonius* are recognized below were also barcoded using a 597bp fragment of cytochrome c oxidase subunit I (COI). The species are: *Allochthonius buanensis* (Lee, 1982 GenBank No. OR290020), *Allochthonius coreanus* (Morikawa, 1970 OR290038), *Allochthonius jungsuni* **sp. nov.** (PX533168), *Allochthonius maximus* **sp. nov.** (PX921017), and *Allochthonius rufimanus* **sp. nov.** (PX533173). *A. buanensis* and *A. coreanus* used in this study were sequenced by Harms et al. (2024), which are sampled in their type localities. DNA was extracted by grinding two or three legs detached from each specimen, using Dneasy Blood & Tissue Kit (QIAGEN, Valencia, CA) and LaboPss^Tm^ DNA purification Kit (Cosmo Genetech Co. Ltd., Seoul, Republic of Korea) following the manufacturer’s protocol. PCR amplification was conducted using the primer set LCO1490 (5′-GGTCAACAAATCATCATAAAGATATTGG-3′) (Folmer et al., 1994) and HCOoutout (5′-GTAAATATATGRT GDGCTC-3′) (Prendini, Weygoldt & Wheeler, 2005) and under the following conditions: initial denaturation at 94 °C for 2min, followed by 40 cycles of denaturation at 95 °C for 30 s, annealing at 40–45 °C for 30 s, extension at 72 °C for 1min, and a final extension at 72 °C for 5min. DNA amplification utilized AccuPower PCR Premix (Bioneer, Daejeon, Republic of Korea) and sequencing was done by Macrogen, Inc. (Geumcheon-Gu, Seoul, Republic of Korea). Interspecific genetic distance was calculated using MEGA 11.0 and the Kimura 2-parameter model (Tamura, Stecher & Kumar, 2021). Sequences generated in this study are deposited in GenBank (Table 1).

**Table 1:** Interspecific genetic divergences within all Korean Allochthonius species.

	*A. buanensis* OR290020	*A. coreanus* OR290038	*A. jungsuni*** sp. nov.** PX533168	*A. maximus*** sp. nov.** PX921017	*A. rufimanus*** sp. nov.** PX533173
*A. buanensis* OR290020					
*A. coreanus* OR290038	16.2%				
*A. jungsuni*** sp. nov.** PX533168	13.2%	16.0%			
*A. maximus*** sp. nov.** PX921017	20.3%	16.2%	19.0%		
*A. rufimanus*** sp. nov.** PX533173	17.9%	17.9%	17%	24.3%	

### Nomenclatural acts

The electronic version of this article in Portable Document Format (PDF) will represent a published work according to the International Commission on Zoological Nomenclature (ICZN), and hence the new names contained in the electronic version are effectively published under that Code from the electronic edition alone. This published work and the nomenclatural acts it contains have been registered in ZooBank, the online registration system for the ICZN. The ZooBank LSIDs (Life Science Identifiers) can be resolved and the associated information viewed through any standard web browser by appending the LSID to the prefix http://zoobank.org/. The LSID for this publication is: urn:lsid:zoobank.org:pub:A53E1EB1-DE99-45D8-B92B-E538649EEB13. The online version of this work is archived and available from the following digital repositories: PeerJ, PubMed Central SCIE and CLOCKSS.

## Result

### Taxonomy

**Table:** 

**Family Pseudotyrannochthoniidae Beier, 1932**
**Genus *Allochthonius*** **Chamberlin, 1929**

**Type species.** *Chthonius opticus* Ellingsen, 1907, by original designation.

**Table:** 

***Allochthonius buanensis* Lee, 1982**
*Allochthonius buanensis*. Lee, 1982: 76-79, figs. 1a-c, 2a-k, table 1; Bang, 1984: 11-15, table 4–5, plate 1-2, 3-2, 3-4, 5-2, 5-4, 7-2, 9-2, 11-2; Hong, 1996: 19-23.
*Allochthonius* (*Allochthonius*) *buanensis*. Lee & Seo, 1995: 456-459, figs. 1a-d, 2a, 3a, table 1; Hong, Kim & Lee, 1996: 174-175; Ohira et al., 2018: 400, 402, 404-405, fig. 3.

**Material examined.** KOREA 2♀, 3♂: Jeollabukdo-Province, Buan-gun, Byeonsan-myeon, Junggye-ri: 35°38′6.22″N, 126°34′28.29″E; 31 May 2024; KH Jeong leg; JBNU-KH129JB (JBNU).

**Diagnosis.** This species is most similar to *Allochthonius exornatus* Gao and Zhang, 2013 from China by the number of marginal teeth on the fixed chelal finger (16–18 in *A. buanensis*, 18 in *A. exornatus*), six coxal spines, and six setae on the cheliceral palm. Both species can be distinguished by the number of setae on the carapace (10-4: 24 in *A. buanensis vs* 10-4: 26 in *A. exornatus*), and the number of marginal teeth on the movable chelal finger (13–17 in *A. buanensis vs* 5–7 in *A. exornatus*).

**Description.**

**Figure 1:**
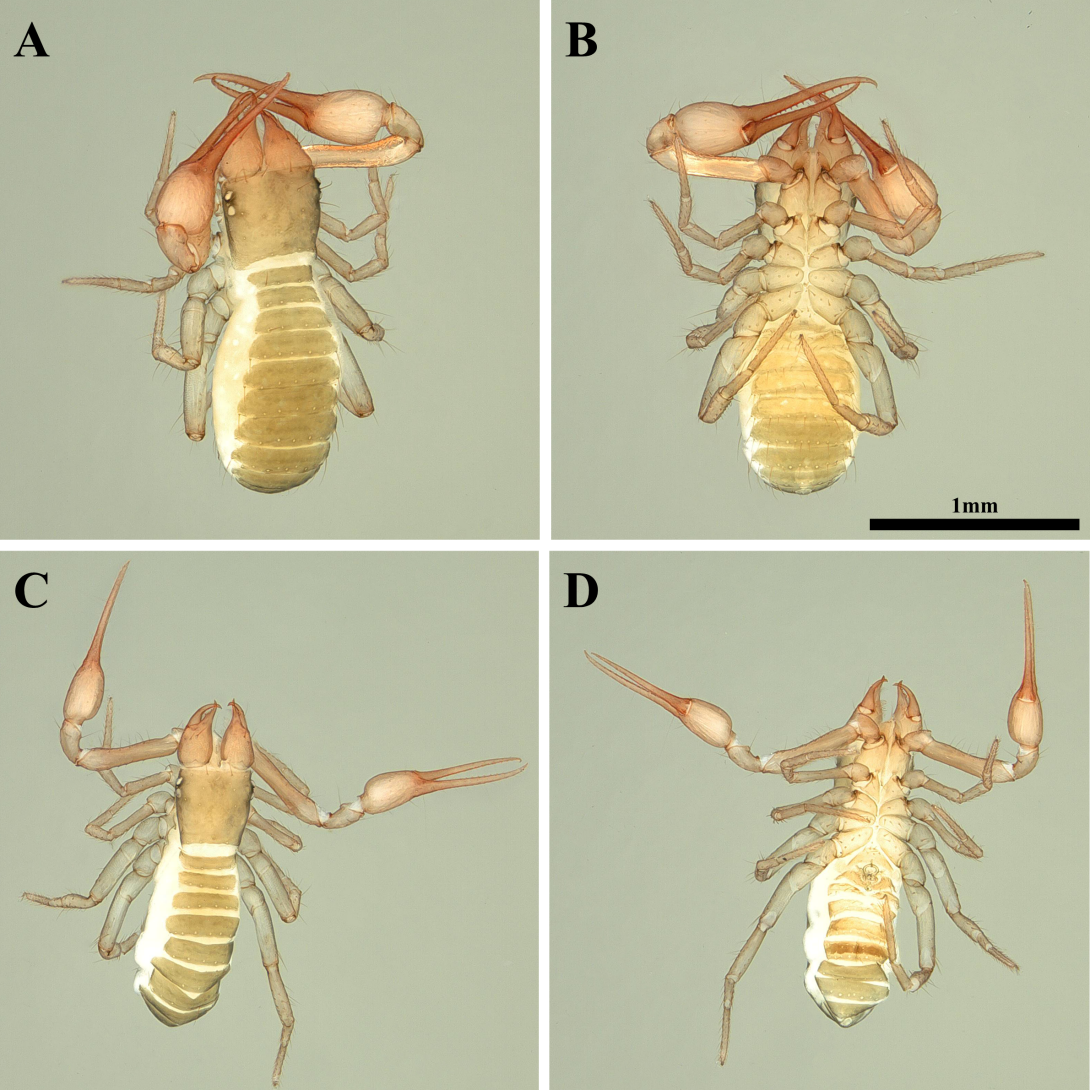
Habitus of *Allochthonius buanensis*Lee, 1982. (A) Female adult, dorsal view; (B) female adult, ventral view; (C) male adult, dorsal view; (D) male adult, ventral view.

**Female, adult (*n* = 2) (Figs. 1A–1B)**

Colour. Body light-brown; pedipalps weakly sclerotized; tip of appendages red.

**Figure 2:**
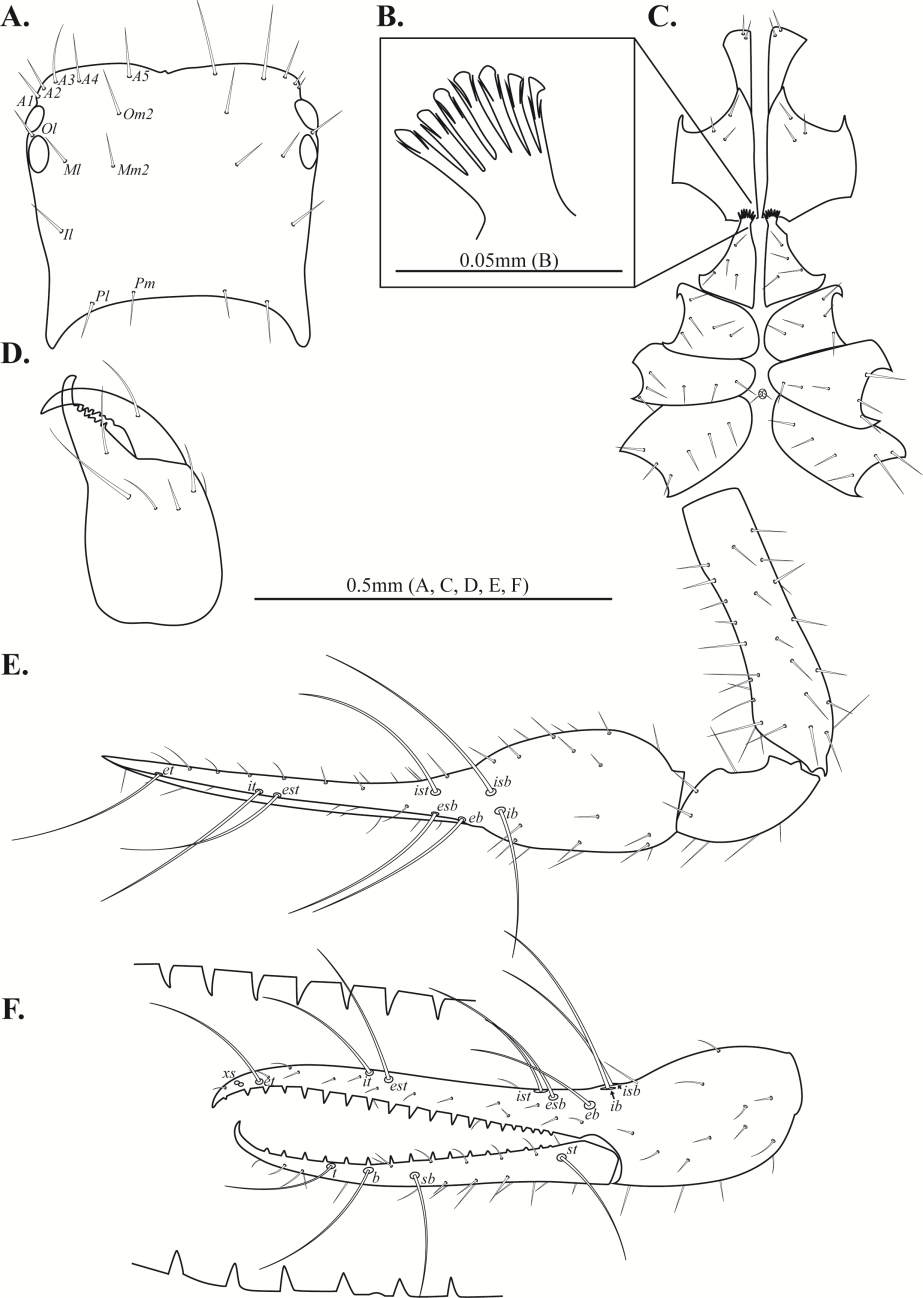
Drawings of *Allochthonius buanensis*Lee, 1982. (A) Carapace, dorsal view; (B) coxal spines; (C) coxa; (D) right chelicera, dorsal view; (E) Left pedipalp, dorsal view; (F) left chela, lateral view.

**Figure 3:**
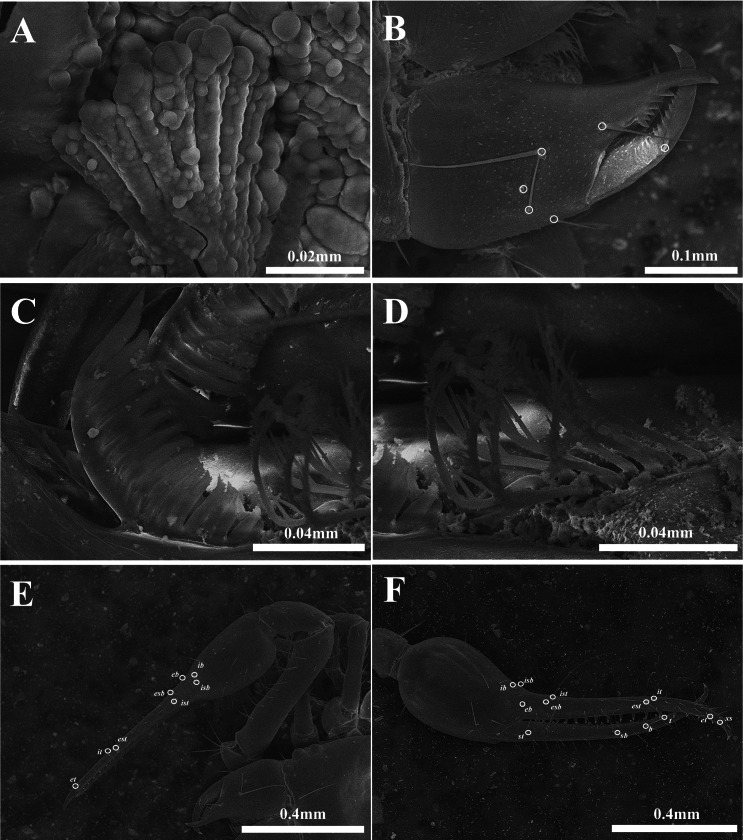
Scanning electron images of *Allochthonius buanensis*Lee, 1982. (A) Coxal spines; (B) right chelicera, dorsal view; (C) serrula exterior; (D) rallum; (E) right pedipalp, dorsal view; (F) left chela, lateral view.

Cephalothorax (Figs. 2A–2C, 3A). Carapace 0.84–0.85 times longer than broad; subquadrate; four distinct eyes; epistome absent; 24 setae on the carapace; 10 setae on the anterior margin (*A1*, *A2*, *A3*, *A4*, *A5*), a pair of setae on the lateral margin (*Ol*), four setae pairs on the medial margin (*Om2*, *Ml*, *Mm2*, *Il*), two pairs of setae on the posterior margin (*Pl*, *Pm*). Two acuminate setae on the manducatory process, maxilla with three setae; coxal chaetotaxy 3–4: 5: 5–6: 6; coxal spines with seven long blades; bisetose intercoxal tubercle present between coxa III and IV.

Chelicera (Figs. 2D, 3B–3D). Palm smooth; five setae on the dorsal of palm, one seta on the ventral of palm, one seta on the movable finger; 5–6 small marginal teeth on the fixed finger, 10–11 marginal teeth on the movable finger; rallum with ten blades; serrula exterior with 16–17 blades.

Pedipalp (Figs. 2E–2F, 3E–3F). Trochanter 1.35–1.37 times, femur 3.74–3.86 times, patella 1.92–1.95 times, chela 3.8–3.89 times, hand 1.46–1.52 times longer than broad, movable finger 1.56–1.6 times longer than hand. Fixed finger with eight trichobothria, movable finger with four trichobothria; *ib* and *isb* positioned basally on the dorsum of the fixed finger; *eb*, *ist*, and *esb* grouped together, *ist* distinctly closer to *esb* than *eb*, *ist* located on the middle of the *eb* and *esb*; *it* and *est* located on the middle of the fixed finger, slightly closer to tip; *et* located next to *xs*, *xs* terminally positioned on the fixed finger; *sb*, *b*, and *t* separately located from *st*; *st* basally positioned on the movable finger. Fixed finger with 17–18, movable finger with 15–16 marginal teeth.

Legs. Leg I: trochanter 1.44 times, femur 3.52–3.68 times, patella 2.97–3.06 times, tibia 2.7–2.71 times, tarsus 7.56–7.61 times longer than broad. Leg IV: trochanter 1.26–1.31 times, femur+patella 3.01–3.03 times, tibia 4.11–4.26 times, metatarsus 2.4–2.7 times, tarsus 6.61–7.16 times longer than broad; arolium undivided; pseudotactile setae basally present on leg IV metatarsus and tarsus.

Abdomen. Pleural membrane granulate; tergites undivided; tergal chaetotaaxy 4: 4: 6: 6–8: 6–7: 6–8: 8–10: 8–10:8–9: 6–7: 4: 0: 0; sternites III–IV divided, sternite V partly divided; sternal chaetotaxy 5–8: 16–18: 13–14: 10–14: 12–14: 10–14: 12–14: 10–14: 8–9: 4: 2.

Dimensions (in mm). Body length 1.45–1.51. Pedipalp: trochanter 0.17–0.18/0.13, femur 0.53–55/0.14–15, patella 0.26–0.27/0.14, chela (with pedicel) 1.01–1.05/0.27, movable finger 0.62–0.64, hand 0.41–0.49/0.27. Chelicera: total 0.41–0.42/0.21, movable finger 0.18–0.2, hand 0.22–0.23/0.21. Carapace 0.41–0.42/0.48–0.49; anterior eye 0.03–0.04; posterior eye 0.03–0.05. Leg I: trochanter 0.20/0.14, femur 0.39–0.40/0.11, patella 0.25/0.08–0.09, tibia 0.19–0.2/0.07, tarsus 0.42–0.43/0.06. Leg IV: trochanter 0.18–0.19/0.14–0.15, femur+patella 0.52–0.53/0.17–0.18, tibia 0.37–0.38/0.09, metatarsus 0.14–0.17/0.06, tarsus 0.27/0.04.

**Male, adult (*n* = 3) (Figs. 1C–1D)**

Cephalothorax. Carapace 0.81–0.93 times longer than broad; with 24 setae; 10 setae on the anterior margin, and four setae on the posterior margin. Coxal chaetotaxy 3–4: 5–6: 5–6: 6–7; coxal spines with seven blades.

Pedipalp. Trochanter 1.38–1.51 times, femur 4.47–5.05 times, patella 1.89–2.19 times, chela 4.66–4.82 times, hand 1.77–1.8 times longer than broad, movable finger 1.6–1.72 times longer than the hand. Fixed finger with 16–18, movable finger with 15–17 marginal teeth.

Legs. Leg I: trochanter 1.12–1.27 times, femur 3.45–4.97 times, patella 2.59–3.03 times, tibia 3.1–3.76 times, tarsus 7.45–8.22 times longer than broad. Leg IV: trochanter 1.19 times, femur+patella 2.54–3.79 times, tibia 4.57–5.6 times, metatarsus 2.6–2.7 times, tarsus 8.73–9.5 times longer than broad.

Abdomen. Tergal chaetotaxy 4: 4: 6: 7: 8: 9: 9: 9: 6: 4: 0: 0; sternal chaetotaxy 7: 28: 12: 15: 15: 16: 14: 10: 2–4: 2.

Dimensions (in mm). Body length 1.58–1.61. Pedipalp: trochanter 0.16–0.17/0.11–0.12, femur 0.51–0.53/0.11, patella 0.20–0.21/0.10, chela (with pedicel) 0.81–0.85/0.17–0.18, movable finger 0.52, hand 0.29–0.33/0.17–0.18. Chelicera: total 0.33–0.37/0.17–0.18, movable finger 0.19, hand 0.14–0.18/0.17–0.18. Carapace 0.34–0.42/0.42–0.45; anterior eye 0.04–0.05; posterior eye 0.05. Leg I: trochanter 0.11–0.12/0.09, femur 0.25–0.30/0.06–0.07, patella 0.14–0.19/0.05–0.06, tibia 0.15–0.17/0.04–0.05, tarsus 0.27–0.32/0.04. Leg IV: trochanter 0.13/0.11, femur+patella 0.44–0.48/0.13–0.17, tibia 0.34–0.46/0.07–0.08, metatarsus 0.14–0.15/0.05–0.06, tarsus 0.34–0.36/0.04.

**Sequence data.** GenBank Accession No. OR290020. *A. buanensis* differs from other Korean species by 13.2% (*A. jungsuni* **sp. nov.**) to 20.3% (*A. maximus* **sp. nov.**) pairwise divergences in the CO1 data respectively.

**Table:** 

***Allochthonius coreanus* Morikawa, 1970**
*Allochthonius* (*Allochthonius*) *opticus coreanus*. Morikawa, 1970: 141-143, , figs. 1, 2a; Lee & Seo, 1995: 460-464, figs. 2b, 3c, 5a-d, 6; Hong, Kim & Lee, 1996: 175.
*Allochthonius coreanus*. Bang, 1984: 6-8, table 2-3, plate 1-1, 3-1, 3-3, 5-1, 5-3, 7-1, 9-1, 11-1; Hong & Kim, 1993: 2; Hong, 1996: 23-24.
*Allochthonius* (*Allochthonius*) *coreanus*. Ohira et al., 2018: 400.

**Materials examined.** KOREA 1♀, 2♂: Gangwondo-Province, Jeongseon-gun, Bukpyeong-myeon, Najeon-ri, 256-1; 37°26′21.4″N 128°37′30.7″E; 23 April 2020; YJ Choi and UJ Byeon leg; JBNU-KH1086GW (JBNU). KOREA 1♀, 3♂: Gyeonggido-Province, Yangpyeong-gun, Yangdong-myeon, Hwanggeo-gil, 262-10; 37°27′39.60″N, 127°43′5.68″E; 14 May 2022; KH Jeong leg; ZMH-A@@ (ZMH; 1♀, 1♂), JBNU-KH028GG (JBNU, 2♂).

**Diagnosis.** This species is most similar to *Allochthonius fanjingshan* Gao, Zhang and Zhang, 2016 in the following characteristics: number of carapacal setae (10-4: 28 in both species) and six setae on the cheliceral palm. It can be distinguished from *A. fanjingshan* by the following characteristics: number of coxal spines (seven in *A. coreanus vs* nine in *A. fanjingshan*), the number of marginal teeth on the chelal fingers (24–30 in fixed finger, 27–31 in movable finger of *A. coreanus vs* 15–16 in fixed finger, 20–21 in movable finger of *A. fanjingshan*).

**Description.**

**Figure 4:**
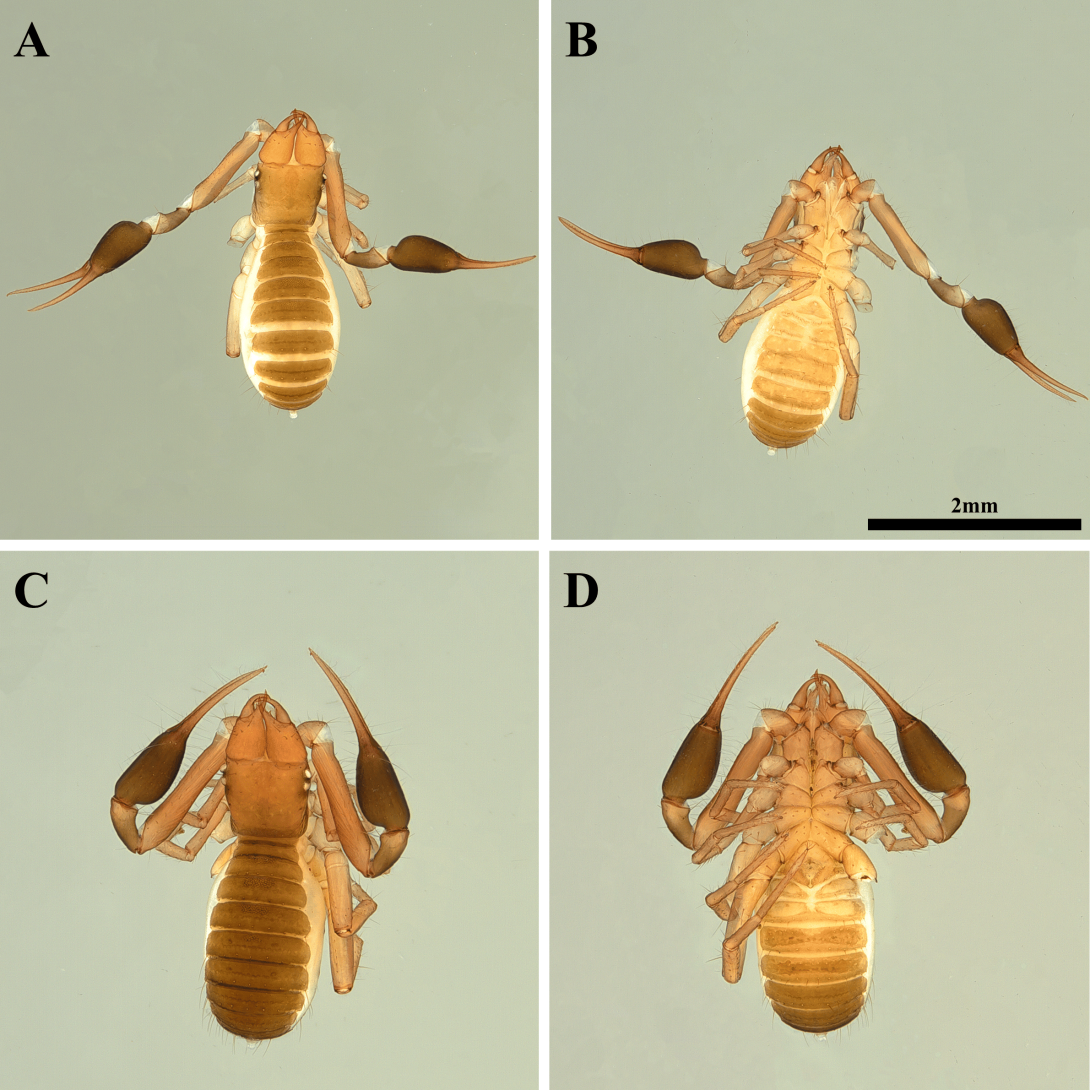
Habitus of *Allochthonius coreanus*Morikawa, 1970. (A) Female adult, dorsal view; (B) female adult, ventral view; (C) m)ale adult, dorsal view; (D) male adult, ventral view.

**Female, adult (*n* = 2) (Figs. 4A–4B)**

Colour. Body dark-brown; pedipalps strongly sclerotized; chela black.

**Figure 5:**
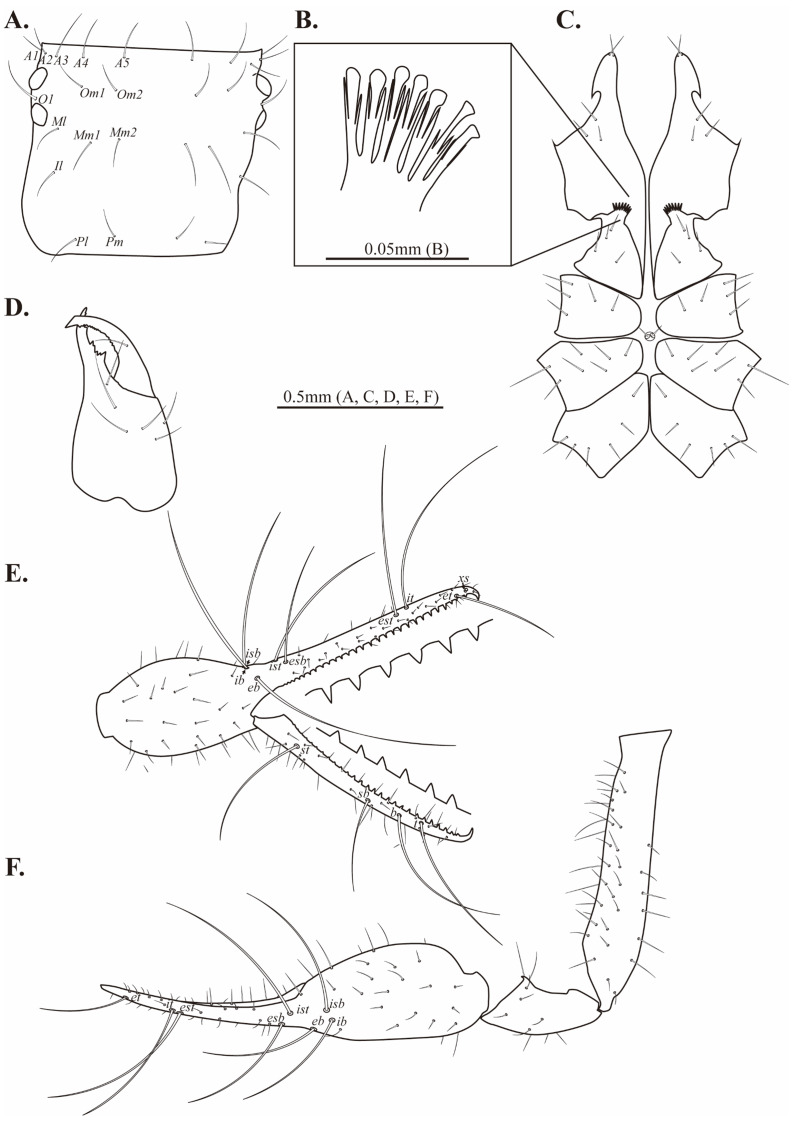
Drawings of *Allochthonius coreanus*Morikawa, 1970. (A) Carapace, dorsal view; (B) coxal spines; (C) coxa; (D) right chelicera, dorsal view; (E) left pedipalp, dorsal view; (F) left chela, lateral view.

**Figure 6:**
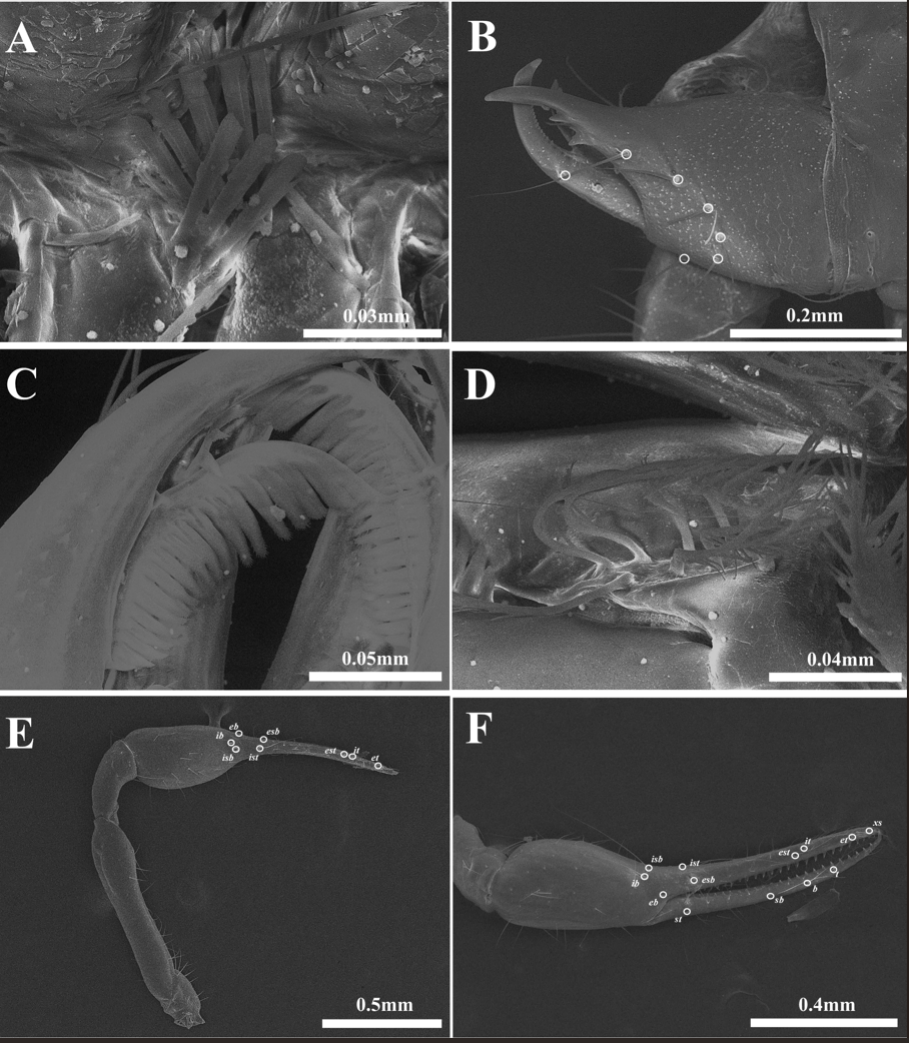
Scanning electron images of *Allochthonius coreanus*Morikawa, 1970. (A) Coxal spines; (B) left chelicera, dorsal view; (C) serrula exterior; (D) rallum; (E) left pedipalp, dorsal view; (F) right chela, lateral view.

Cephalothorax (Figs. 5A–5C, 6A). Carapace 0.88–0.96 times longer than broad; rectangular; four distinct eyes; epistome absent; 28 setae on the carapace; five setae pairs on the anterior margin (*A1*, *A2*, *A3*, *A4*, *A5*), a pair of setae on the lateral margin (*Ol*), six setae pairs on the medial margin (*Om1*, *Om2*, *Ml*, *Mm1*, *Mm2*, *Il*), and two pairs of setae on the posterior margin (*Pl*, *Pm*). Two acuminate setae on the manducatory process, maxilla with three setae; coxal chaetotaxy 3–4: 3–5: 6–7: 5–7; coxal spines with seven long blades; bisetose intercoxal tubercle present between coxa III and IV.

Chelicera (Figs. 5D, 6B–6D). Palm smooth; palm with six setae, one seta on the movable finger; 3–5 large marginal teeth on the fixed finger, 11–14 small marginal teeth on the movable finger; rallum with eight blades; serrula exterior with 18–19 blades.

Pedipalp (Figs. 5E–5F, 6E–6F). Trochanter 1.32–1.44 times, femur 4.5–4.93 times, patella 1.69–2.2 times, chela 4.14–4.65 times, hand 1.83–1.88 times longer than broad, movable finger 1.27–1.47 times longer than the hand. Fixed finger with 28–30, movable finger with 27–30 small and dentate marginal teeth. Fixed finger with eight trichobothria, movable finger with four trichobothria; *ib* and *isb* positioned basally on the dorsum of the fixed finger; *eb*, *ist*, and *esb* grouped together, *ist* located distinctly closer to *esb* than *eb*; *it* and *est* located on the middle of the fixed finger, closer to tip; *et* located next to *xs*, *xs* positioned terminally on the fixed finger; *sb*, *b*, and *t* separately located from *st*; *st* basally positioned on the movable finger.

Legs. Leg I: trochanter 1.32–1.47 times, femur 3.82–5.78 times, patella 2.76–3.78 times, tibia 2.87–3.63 times, tarsus 7.17–8 times longer than broad. Leg IV: trochanter 1.33–1.78 times, femur+patella 2.66–2.9 times, tibia 3.31–4.92 times, metatarsus 2.85–3.3 times, tarsus 6.44–9.5 times longer than broad; arolium undivided; pseudotactile setae basally present on leg IV metatarsus and tarsus.

Abdomen. Pleural membrane granulate; tergites undivided; tergal chaetotaxy 4: 6–7: 7–8: 8–9: 8–9: 10–11: 10–12: 11: 11: 10: 0: 0; sternites III–IV divided, sternite V partly divided; sternal chaetotaxy 10–12: 18–22: 16–18: 14: 14: 10–14: 10–11: 9–12: 8–11: 2: 2.

Dimensions (in mm). Body length 2.01–2.43. Pedipalp: trochanter 0.24–0.29/0.17–0.22, femur 0.83–0.9/0.17–0.2, patella 0.32–0.44/0.14–0.2, chela (with pedicel) 1.22–1.45/0.26–0.35, movable finger 0.73–0.81, hand 0.49–0.64/0.26–0.35. Chelicera: total 0.51–0.6/0.24–0.3, movable finger 0.26–0.32, hand 0.25–0.28/0.24–0.3. Carapace 0.51–0.58/0.53–0.66; anterior eye 0.06; posterior eye 0.05–0.07. Leg I: trochanter 0.19–0.25/0.14–0.17, femur 0.42–0.45/0.08–0.11, patella 0.25–0.34/0.09, tibia 0.22–0.29/0.08, tarsus 0.42–0.56/0.06–0.07. Leg IV: trochanter 0.30–0.32/0.18–0.2, femur+patella 0.60–0.87/0.23–0.3, tibia 0.48–0.64/0.13–0.15, metatarsus 0.23–0.33/0.08–0.1, tarsus 0.34–0.57/0.05–0.06.

**Male, adult (*n* = 5) (Figs. 4C–4D)**

Cephalothorax. Carapace 0.92–0.99 times longer than broad and with 28 setae; 10 setae on the anterior margin, and four setae on the posterior margin. Coxal chaetotaxy 4: 4–5: 6–8: 5–8. Coxal spines with eight blades.

Pedipalp. Trochanter 1.35–1.44 times, femur 4.7–4.94 times, patella 1.69–2.05 times, chela 4.5–4.65 times, hand 1.88–1.97 times longer than broad, movable finger 1.29–1.47 times longer than the hand. Fixed finger with 24–27, movable finger with 28–31 marginal teeth.

Legs. Leg I: trochanter 1.28–1.32 times, femur 3.64–5.78 times, patella 2.76–3.27 times, tibia 2.87–3.38 times, tarsus 7.17–8.5 times longer than broad. Leg IV: trochanter 1.33–1.71 times, femur+patella 2.66–3.12 times, tibia 3.31–3.69 times, metatarsus 2.85–3.11 times, tarsus 6.44–8.33 times longer than broad.

Abdomen. Tergal chaetotaxy 4: 6–8: 8–9: 8–9: 8–9: 10–12: 10–12: 9–11: 10–11: 10: 0: 0; sternal chaetotaxy 10–12: 36–40: 16–20: 14–18: 15–16: 10–12: 10–11: 9–10: 8–10: 2–4: 2.

Dimensions (in mm). Body length 1.83–2.41. Pedipalp: trochanter 0.24–0.27/0.17–0.2, femur 0.83–0.94/0.17–0.2, patella 0.32–0.43/0.19–0.21, chela (with pedicel) 1.22–1.44/0.26–0.32, movable finger 0.73–0.81, hand 0.5–0.63/0.26–0.32. Chelicera: total 0.51–0.56/0.24–0.26, movable finger 0.26–0.29, hand 0.25–0.27/0.24–0.26. Carapace 0.50–0.56/0.50–0.61; anterior eye 0.05–0.06; posterior eye 0.04–0.06. Leg I: trochanter 0.19–0.23/0.14–0.18, femur 0.4–0.45/0.08–0.11, patella 0.25–0.36/0.09–0.11, tibia 0.22–0.27/0.08, tarsus 0.42–0.51/0.06. Leg IV: trochanter 0.20–0.29/0.15–0.17, femur+patella 0.61–0.81/0.23–0.26, tibia 0.48/0.13–0.15, metatarsus 0.23–0.28/0.08–0.09, tarsus 0.34–0.5/0.05–0.06.

**Sequence data.** GenBank Accession No. OR290038. *A. coreanus* differs from the other Korean species by 16% (*A. jungsuni* **sp. nov.**) to 17.9% (*A. rufimanus* **sp. nov.**) pairwise divergence in the CO1 data respectively.

**Table:** 

***Allochthonius jungsuni* Jeong and Harms sp. nov.**

**LSID:** urn:lsid:zoobank.org:act:EA291957-FD99-4772-8DD1-C7E566C242D8

**Type material.** Holotype: KOREA ♀: Gyeongsangnamdo-Province, Geoje-si, Dongbu-myeon, Gojejungang-ro, 325; 34°46′59.54″N, 128°37′33.06″E; 18 April 2024; KH Jeong and Y Cha leg; NIBRIV0000932089 (NIBR). Paratypes 3♂; the same data as the holotype; NIBRIV0000932089 (NIBR; 1♂), JBNU-KH107GN-A (JBNU, 2♂).

**Etymology.** The specific epithet is dedicated to Dr. Jung-Sun Yoo in recognition of his sincere support, which substantially contributed to the first author’s research in arachnology.

**Diagnosis.** This species differs from other congeners by the number of coxal spines. Whereas species of *Allochthonius* typically possess fewer than ten coxal spines, this species has 12–13 spines on coxa I. This species is most similar to *Allochthonius borealis* Sato, 1984 and shares the same number of carapacal setae (10-4: 26 in both species) and marginal teeth on the fixed chelal finger (18–20 in *A. borealis*, 19 in *A. jungsuni* **sp. nov.**). Both species can easily be distinguished by the number of marginal teeth on the movable chelal finger (8–9 in *A. borealis vs* 28 in *A. jungsuni* **sp. nov.**) and the number of setae on the cheliceral palm (5 in *A. borealis vs* 7 in *A. jungsuni* **sp. nov.**).

**Description.**

**Figure 7:**
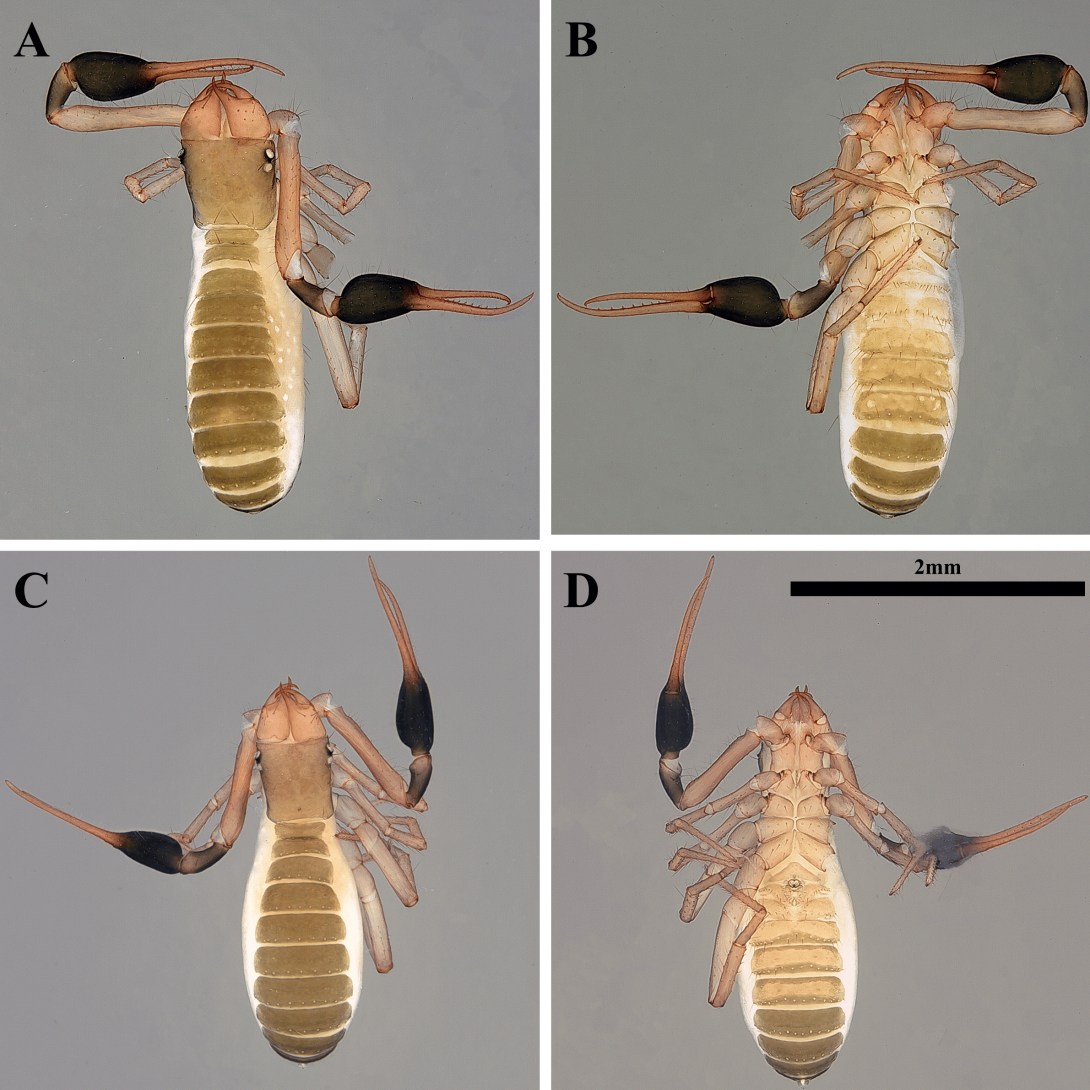
Habitus of *Allochthonius jungsuni* sp. nov. (A) Female adult, dorsal view; (B) female adult, ventral view; (C) male adult, dorsal view; (D) male adult, ventral view.

**Holotype, female, adult (Figs. 7A–7B)**

Colour. Body brownish-grey; pedipalp strongly sclerotized; chelal palm and pedipalpal patella black; chelal finger red.

**Figure 8:**
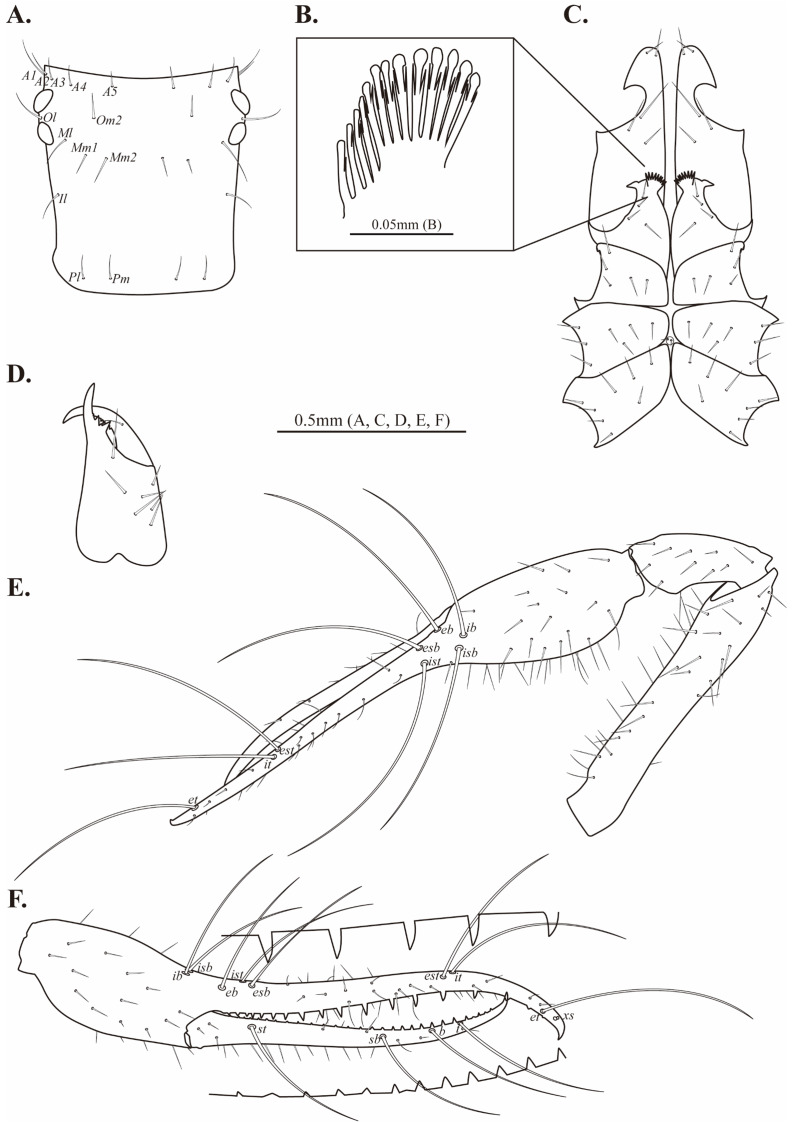
Drawings of *Allochthonius jungsuni* sp. nov. (A) Carapace, dorsal view; (B) coxal spines; (C) coxa; (D) right chelicera, dorsal view; (E) right pedipalp, dorsal view; (F) right chela, lateral view.

**Figure 9:**
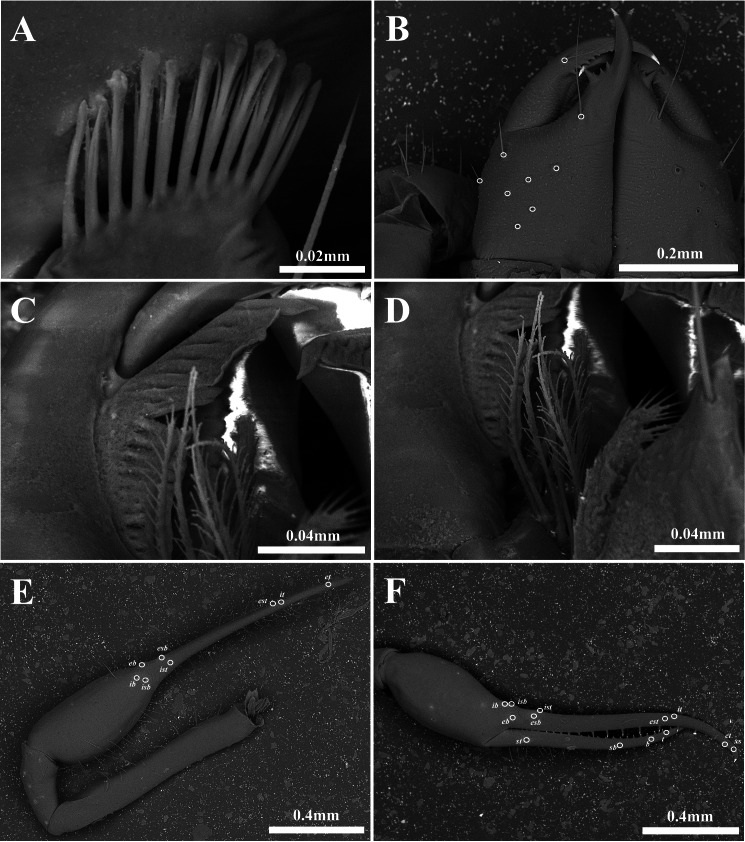
Scanning electron images of *Allochthonius jungsuni* sp. nov. (A) Coxal spines; (B) left chelicera, dorsal view; (C) serrula exterior; (D) rallum; (E) left pedipalp, dorsal view; (F) left chela, lateral view.

Cephalothorax (Figs. 8A–8C, 9A). Carapace 0.97 times longer than broad; rectangular; four distinct eyes; epistome absent; 26 setae on the carapace; five setae pairs on the anterior margin (*A1*, *A2*, *A3*, *A4*, A5*),* a pair of setae on the lateral margin (*Ol*), five pairs of setae on the medial margin (*Om2*, *Ml*, *Mm1*, *Mm2*, *Il*), two setae pairs on the posterior margin (*Pl*, *Pm*). Two acuminate setae on the manducatory process, maxilla with three setae; coxal chaetotaxy 4: 5: 7: 8; coxal spines with 13 long blades; bisetose intercoxal tubercle present between coxa III and IV.

Chelicera (Figs. 8D, 9B–9D). Palm smooth and with seven setae, one seta on the movable finger; five marginal teeth on the fixed finger, two large teeth positioned basally on the fixed finger; eight small marginal teeth on the movable finger; rallum with eight blades; serrula exterior with 20 blades.

Pedipalp (Figs. 8E–8F, 9E–9F). Trochanter 1.44 times, femur 5.57 times, patella 2 times, chela 4.5 times, hand 2.36 times longer than broad, movable finger 0.91 times longer than hand. Fingers strongly curved. Fixed finger with eight trichobothria, movable finger with four trichobothria; *isb* and *ib* basally positioned on the dorsum of the fixed finger; *eb*, *ist*, and *esb* grouped together, *ist* positioned distinctly closer to *esb* than *eb*; *est* and *it* positioned on the middle of the fixed finger, located closer to tip than the basal of the fixed finger; *et* positioned next to *xs*; *xs* located terminally on the fixed finger; *sb*, *b*, and *t* positioned apart from *st*; *st* position basally on the movable finger. Fixed finger with 19, movable finger with 28 marginal teeth; strong and large marginal teeth on the fixed finger, small and undeveloped teeth on the fixed finger.

Legs. Leg I: trochanter 1.29 times, femur 5.44 times, patella 3.88 times, tibia 4.04 times, tarsus 7.81 times longer than broad. Leg IV: trochanter 1.73 times, femur+patella 4.06 times, tibia 5.14 times, metatarsus 3.5 times, tarsus 10.33 times longer than broad.

Abdomen. Tergites undivided; tergal chaetotaxy 4: 6: 7: 9: 10: 10: 10: 11: 8: 6: 0: 0; sternite III partly divided, sternite IV divided; sternal chaetotaxy 10: 28: 18: 15: 14: 14: 16: 12: 10: 2: 2.

Dimensions (in mm). Body length 2.82. Pedipalp: trochanter 0.26/0.18, femur 1.05/0.19, patella 0.45/0.22, chela (with pedicel) 1.68/0.37, movable finger 0.8, hand 0.88/0.37. Chelicera: total 0.59/0.28, movable finger 0.34, hand 0.25/0.28. Carapace 0.69/0.71; anterior eye 0.07; posterior eye 0.06. Leg I: trochanter 0.23/0.18, femur 0.56/0.1, patella 0.39/0.1, tibia 0.3/0.07, tarsus 0.52/0.07. Leg IV: trochanter 0.35/0.2, femur+patella 0.95/0.23, tibia 0.63/0.12, metatarsus 0.29/0.08, tarsus 0.56/0.05.

**Paratypes, male, adult (*n* = 3) (Figs. 7C–7D)**

Cephalothorax. Carapace 1.03–1.14 times longer than broad and with 26 setae; 10 setae on the anterior margin, four setae on the posterior margin. Coxal chaetotaxy 3–4: 4–6: 5–7: 7–8; coxa I with 12–13 short blades.

Pedipalp. Trochanter 1.4–1.44 times, femur 4.92–5.79 times, patella 2.08–2.23 times, chela 5.49–5.56 times, hand 2.4–2.64 times longer than broad, movable finger 1.08–1.31 times longer than the hand. Movable finger with 16–19, fixed finger 27–30 with marginal teeth.

Legs. Leg I: trochanter 1.26–1.39 times, femur 4.01–5.22 times, patella 3.71–3.84 times, tibia 4–4.93 times, tarsus 8.73–9.61 times longer than broad. Leg IV: trochanter 1.61–1.72 times, femur+patella 3.71–3.8 times, tibia 5.68–5.03 times, metatarsus 3.85–3.87 times, tarsus 7.44–10.29 times longer than broad.

Abdomen. Tergal chaetotaxy 4: 5–6: 7–8: 7–10: 9–10: 9–10: 11–12: 10–11: 8: 6: 0: 0; sternal chaetotaxy 10: 36–40: 16–18: 15–16: 13–16: 13–14: 14:12: 8: 2: 2.

Dimensions (in mm). Body length 2.58–2.79. Pedipalp: trochanter 0.27–0.28/0.19–0.20, femur 0.88–1.04/0.17–0.19, patella 0.35–0.42/0.19–0.2, chela (with pedicel) 1.56–1.6/0.27–0.29, movable finger 0.75–0.89, hand 0.68–0.85/0.27–0.28. Chelicera: total 0.5–0.52/0.24–0.25, movable finger 0.25–0.3, hand 0.2–0.27/0.24–0.25. Carapace 0.6–0.68/0.59–0.61; anterior eye 0.07–0.08; posterior eye 0.07–0.08. Leg I: trochanter 0.2–0.22/0.15–0.18, femur 0.43–0.57/0.11, patella 0.34–0.38/0.09–0.1, tibia 0.29–0.33/0.07–0.08, tarsus 0.56–0.64/0.06–0.07. Leg IV: trochanter 0.3–0.35/0.17–0.22, femur+patella 0.86–0.93/0.25, tibia 0.62–0.68/0.12–0.13, metatarsus 0.28–0.35/0.09–0.1, tarsus 0.5–0.67/0.6–0.07.

**Sequence data.** GenBank Accession No. PX533168. It differs from other Korean species by 13.2% (*A. buanensis*) to 19% (*A. maximus* **sp. nov.**) pairwise divergences in the CO1 data respectively.

**Table:** 

***Allochthonius maximus* Jeong and Harms sp. nov.**

**LSID:** urn:lsid:zoobank.org:act:3C5116C3-D9BB-4B2B-90F1-777F0DC48554

**Type material.** Holotype: Korea ♂; Gyeongsangbukdo-Province, Yeongcheon-si, Hwabuk-myeon, Janggak-ri, 1121; 36°07′45.29″N, 128°16′18.31″E; 22 May. 2023; JH Oh leg; NIBRIV0000932087 (NIBR). Paratypes 2♀; the same data as the holotype; NIBRIV0000932087 (NIBR; 1♀), JBNU-JH25B (JBNU, 1♀).

**Etymology.** The specific epithet *maximus* refers to its status as the largest epigean species within *Allochthonius*, reaching a body length of approximately three mm.

**Diagnosis.** This species closely resembles *Allochthonius coreanus* (Morikawa, 1970) in its relatively large body size (2.01–2.43 mm in *A. coreanus*, 2.59–3.03 mm in *A*. *maximus* **sp. nov.**) and the number of marginal teeth on the fixed chelal teeth (24–30 in *A. coreanus*, 25–26 in *A*. *maximus* **sp. nov.**), and the presence of six setae on the cheliceral palm. Both species differ in the number of marginal teeth on the movable finger (27–31 in *A. coreanus vs* 22–23 in *A*. *maximus* **sp. nov.**), the number of carapacal setae (10-4: 28 in *A. coreanus vs* 10-4: 26 in *A*. *maximus* **sp. nov.**), and the shape of female’s chelal finger (normal in *A. coreanus vs* strongly curved in *A. maximus* **sp. nov.**).

**Description.**

**Figure 10:**
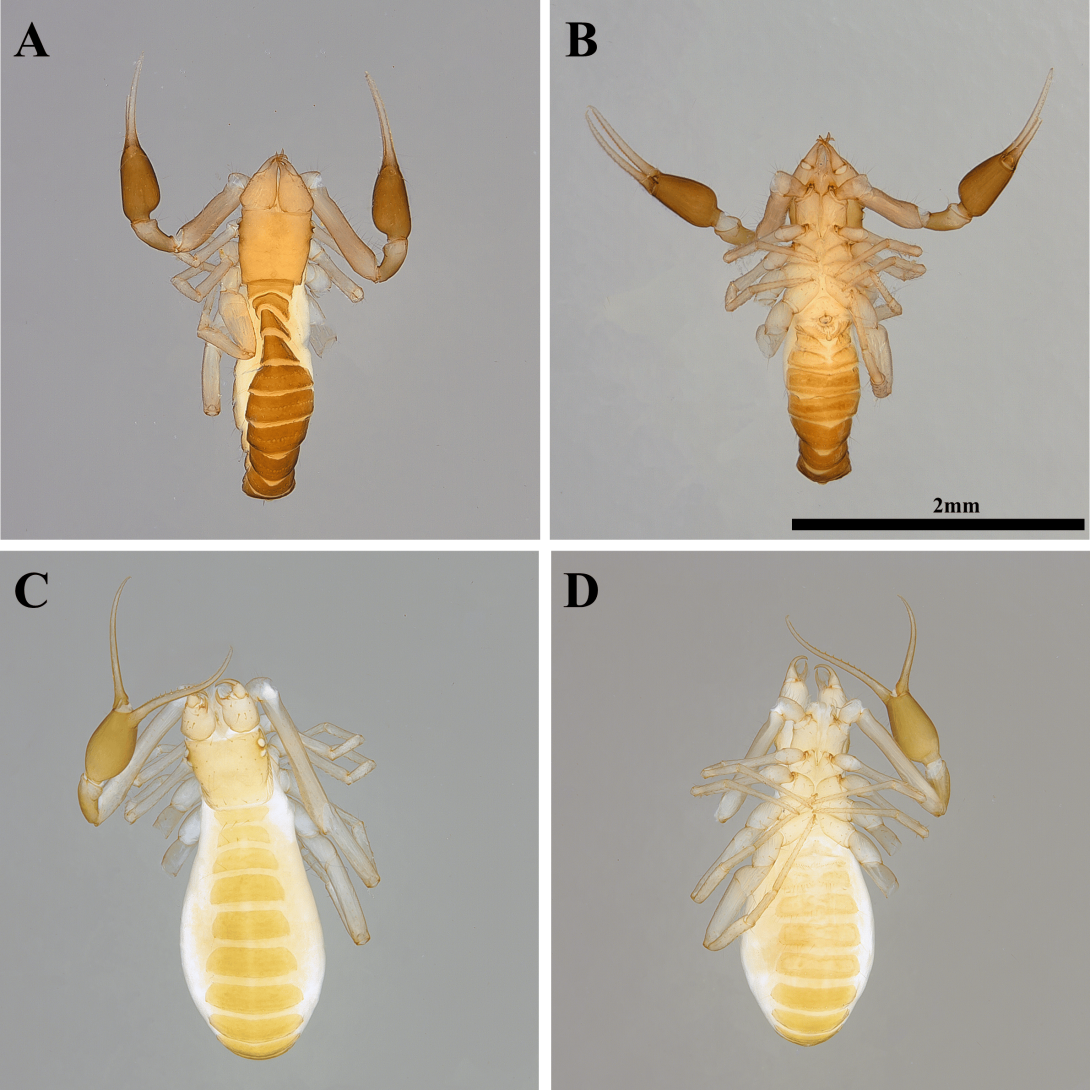
Habitus of *Allochthonius maximus* sp. nov. (A) Male adult, dorsal view; (B) male adult, ventral view; (C) female adult, dorsal view; (D) female adult, ventral view.

**Holotype, male, adult (Figs. 10A–10B)**

Colour. Male body dark brown; pedipalps strongly sclerotized.

**Figure 11:**
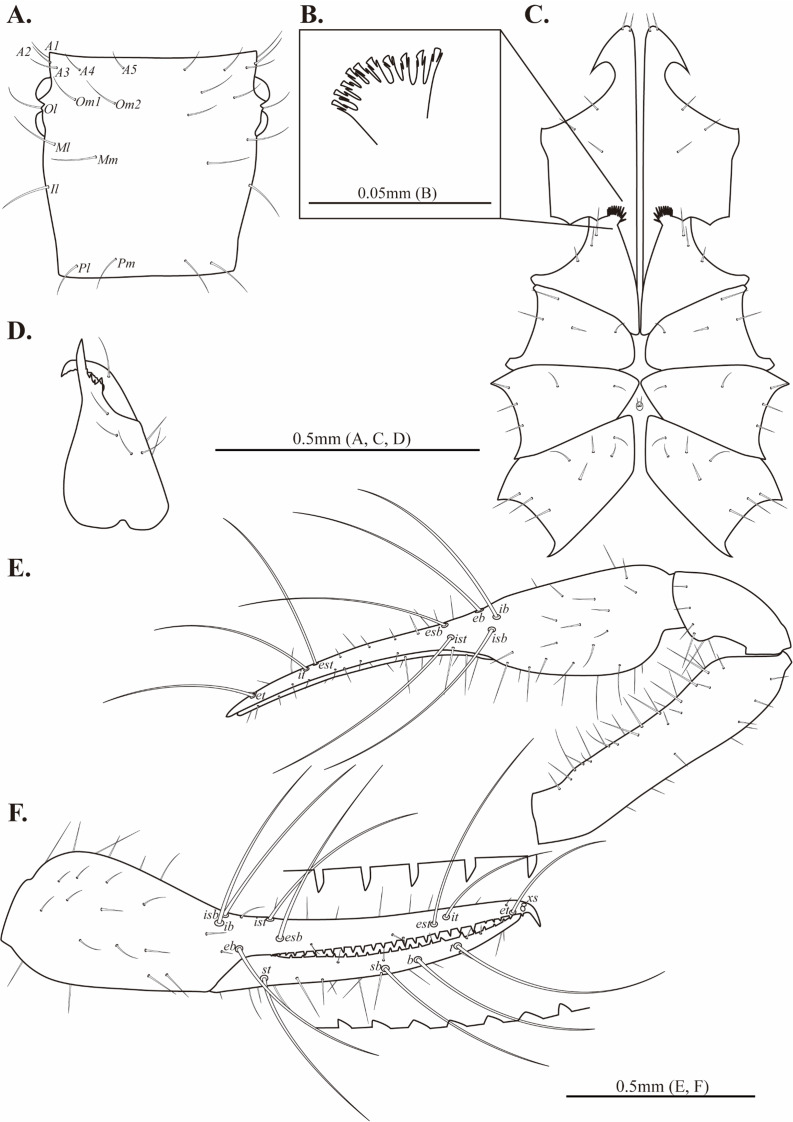
Drawings of *Allochthonius maximus* sp. nov. (A) Carapace, dorsal view; (B) coxal spines; (C) coxa; (D) rightchelicera, dorsal view; (E) right pedipalp, dorsal view; (F) right chela, lateral view.

**Figure 12:**
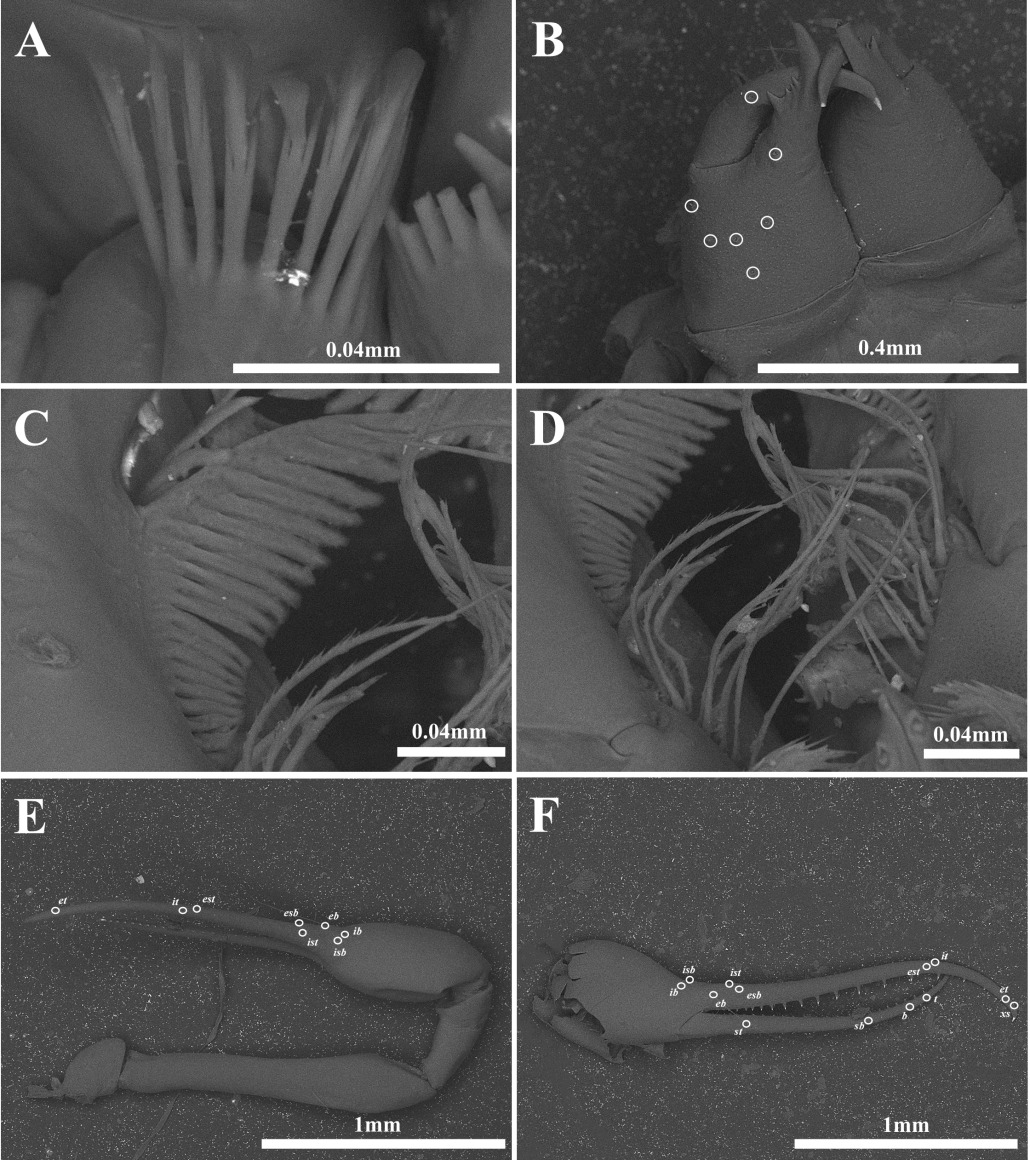
Scanning electron images of *Allochthonius maximus* sp. nov. (A) Coxal spines; (B) left chelicera, dorsal view; (C) serrula exterior; (D) rallum; (E) right pedipalp, dorsal view; (F) right chela, lateral view.

Cephalothorax (Figs. 11A–11C, 12A). Carapace 0.93 times longer than broad; rectangular; four distinct eyes; epistome absent; carapace with 26 setae, five pairs on the anterior margin (*A1*, *A2*, *A3*, *A4*, *A5*), a pair of seate on the lateral margin (*Ol*), five pairs on the medial margin (*Om1*, *Om2*, *Ml*, *Mm*, *Il*), two setae pairs on the posterior margin (*Pl*, *Pm*). Two acuminate setae on the manducatory process, maxilla with three setae; coxal chaetotaxy 3: 4: 5: 7; coxal spines with ten short blades on a common mound; bisetose intercoxal tubercle present between coxa III and IV.

Chelicera (Figs. 11D, 12B–12D). Palm smooth and with six setae, one seta on the movable finger; four marginal teeth on the fixed finger, large tooth basally positioned on the fixed finger; 11 marginal teeth on the movable finger; rallum with 10 blades; serrula exterior with 21 blades.

**Figure 13:**
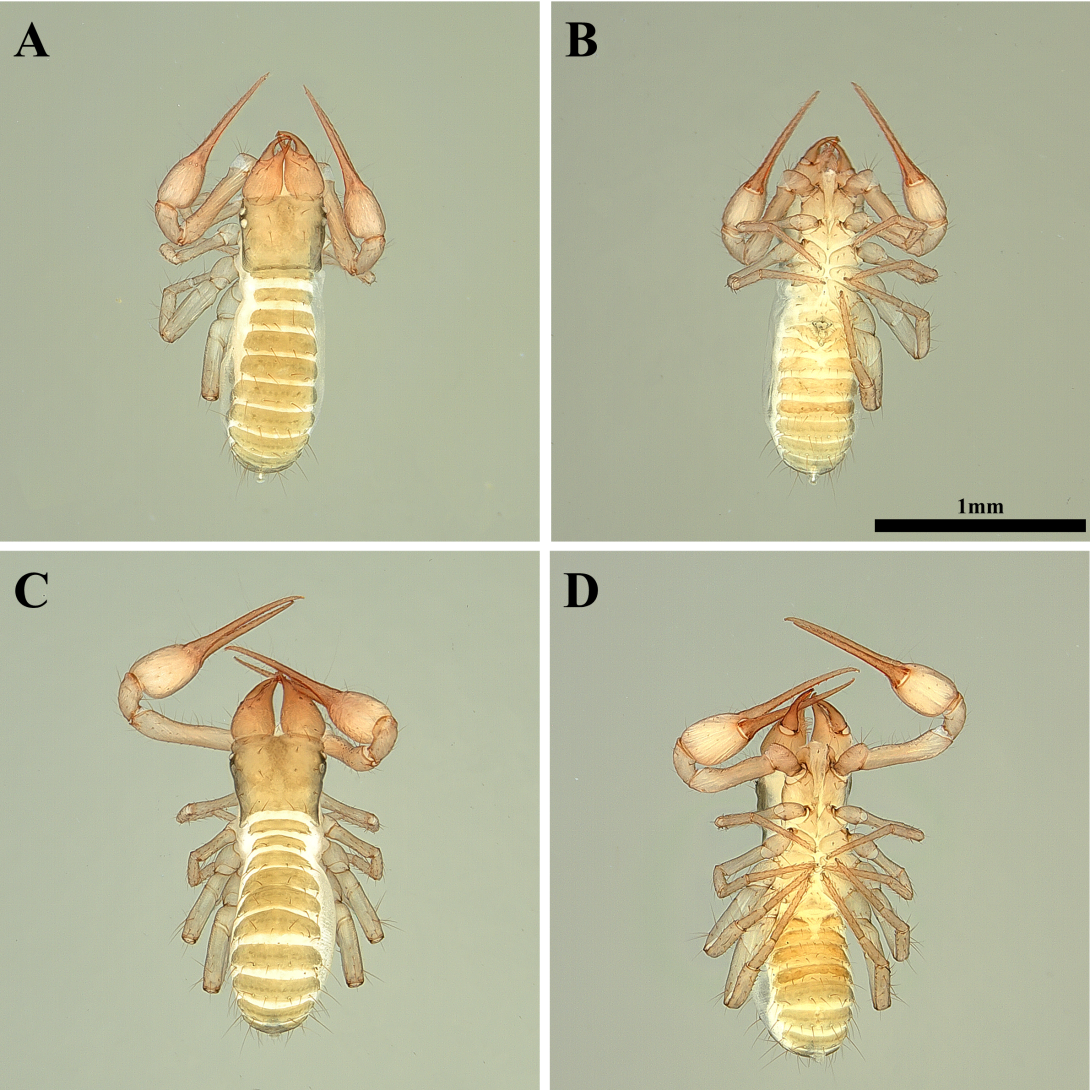
Habitus of *Allochthonius rufimanus* sp. nov. (A) Male adult, dorsal view; (B) male adult, ventral view; (C) female adult, dorsal view; (D) female adult, ventral view.

Pedipalp (Figs. 12B–12F, 13E–13F). Trochanter 1.42 times, femur 4.16 times, patella 1.85 times, chela 4.92 times, hand 2.01 times longer than broad, movable finger 2.91 times longer than the hand. Fixed finger with eight trichobothria, movable finger with four trichobothria; *isb* and *ib* basally positioned on the dorsum of the fixed finger; *eb*, *ist*, and *esb* grouped together, *ist* positioned closer to *esb* than *eb*; *est* and *it* located on the middle of the fixed finger, closer to tip; *et* positioned next to *xs*; *xs* terminally positioned on the fixed finger; *sb*, *b*, and *t* separately located from *st*; *st* basally positioned on the movable finger. Fixed finger with 25–24, movable finger 20 marginal teeth.

Legs. Leg I: trochanter 1.73 times, femur 3.75 times, patella 2.95 times, tibia 3.62 times, tarsus 9.38 times longer than broad. Leg IV: trochanter 1.69 times, femur+patella 2.75 times, tibia 4.46 times, metatarsus 3.11 times, tarsus 6.68 times longer than broad; arolium undivided.

Abdomen. Pleural membrane granulate; tergites undivided; tergal chaetotaxy 4: 6: 6: 9: 10: 10: 12: 10: 8: 6: 0: 0; sternites III–V divided; sternal chaetotaxy 8: 28: 16: 18: 16: 12: 10: 8: 6: 2: 2.

Dimensions (in mm). Body length 2.15. Pedipalp: trochanter 0.27/0.19, femur 0.79/0.19, patella 0.36/0.19, chela (with pedicel) 1.27/0.26, movable finger 0.75, hand 0.52/0.26. Chelicera: total 0.52/0.24, movable finger 0.28, hand 0.24/0.24. Carapace 0.52/0.56; anterior eye 0.06; posterior eye 0.05. Leg I: trochanter 0.23/0.13, femur 0.44/0.12, patella 0.32/0.11, tibia 0.29/0.08, tarsus 0.6/0.06. Leg IV: trochanter 0.28/0.17, femur+patella 0.73/0.27, tibia 0.55/0.12, metatarsus 0.26/0.09, tarsus 0.41/0.06.

**Paratypes, female, adult (*n* = 2) (Figs. 10C–10D)**

Colour. Female body pale brown; pedipalps strongly sclerotized.

Cephalothorax. Carapace 0.98 times longer than broad; with 26 setae; 10 setae on the anterior margin, and four setae on the posterior margin. Coxal chaetotaxy 3: 4–5: 4–5: 7–8; coxal spines with 10–11 blades.

Pedipalp. Trochanter 1.46–1.48 times, femur 5.45–6.2 times, patella 2.12–2.23 times, chela 4.37–6.12 times, hand 1.73–1.92 times longer than broad, movable finger 1.52–2.18 times longer than the hand. Fixed finger with 25–26, movable finger 22–23 marginal teeth. Both fingers strongly curved.

Legs. Leg I: trochanter 1.1 times, femur 6.28 times, patella 4.34 times, tibia 4.75 times, tarsus 10.26 times longer than broad. Leg IV: trochanter 1.66 times, femur+patella 4.3 times, tibia 6.01 times, metatarsus 4 times, tarsus 9.9 times longer than broad.

Abdomen. Tergites undivided; tergal chaetotaxy 4: 6: 6: 8–9: 9: 9–10: 12: 10: 8–9: 6: 0: 0; sternites III–IV divided, sternite V partly divided; sternal chaetotaxy 10–11: 28: 16–20: 16: 16: 14: 12: 12: 10: 2: 2.

Dimensions (in mm). Body length 2.59–3.03. Pedipalp: trochanter 0.31–0.33/0.21–0.23, femur 1.34–1.35/0.22–0.25, patella 0.50–0.54/0.24–0.25, chela (with pedicel) 1.72–2.02/0.33–0.39, movable finger 1.04–1.39, hand 0.63–0.68/0.33–0.39. Chelicera: total 0.60–0.61/0.26–0.29, movable finger 0.29–0.31, hand 0.3/0.29–0.31. Carapace 0.67–0.69/0.68–0.7; anterior eye 0.06–0.07; posterior eye 0.07. Leg I: trochanter 0.22–0.26/0.19–0.2, femur 0.64–0.7/0.11–0.13, patella 0.43–0.48/0.1–0.11, tibia 0.37–0.38/0.08–0.09, tarsus 0.73–0.74/0.07–0.08. Leg IV: trochanter 0.33–0.35/0.21, femur+patella 1.10–1.15/0.27–0.28, tibia 0.71–0.81/0.13–0.14, metatarsus 0.37/0.09, tarsus 0.7–0.75/0.07.

Sequence data. GenBank Accession No. PX921017. It differs from other Korean species by 16.2% (*A. coreanus*) to 24.3% (*A. rufimanus* **sp. nov.**) pairwise divergences in the CO1 data respectively.

**Table:** 

***Allochthonius opticus* (Ellingsen, 1907)**

**Remarks.** The Korean record of this species by Lee & Seo (1995) is considered a misidentification and does not represent *A. opticus* **s. str**. The original type locality of *A. opticus* is Okayama in Japan (Ellingsen, 1907) but all recent taxonomic studies on *Allochthonius* have pointed to very small distribution ranges, short-range endemism, and poor dispersal capacities in this genus (Lin et al., 2025) and other pseudotyrannochthoniids (You et al., 2023; (Jeong et al., 2025; Harms et al., 2025). Considering the molecular taxonomic and ecological data now available for the genus, it is likely that the range of *A. opticus* is restricted to the Okayama Prefecture on Honshu Island, Japan. For details, see discussion (5. 1. Taxonomy of *Allochthonius* in South Korea).

**Table:** 

***Allochthonius rufimanus* Jeong and Harms sp. nov.**

**LSID:** urn:lsid:zoobank.org:act:9C6953B6-1649-4129-A09F-D5501270B933

**Type material.** Holotype: KOREA ♂; Gyeongsangnamdo-Province, Sacheon-si, Sanam-myeon, Gacheon-ri, 333-1; 35°0′2.76″N, 128°7′33.17″E; 31 Mar 2024; KH Jeong and J Park leg; NIBRIV0000932088 (NIBR). Paratypes 9♀, 12♂; the same data as the holotype; NIBRIV0000932088 (NIBR; 1♀), ZMH-A@@ (ZMH; 3♀, 3♂), JBNU-KH100GN-A (JBNU, 5♀, 8♂).

**Etymology.** The specific epithet *rufimanus* is derived from the Latin *rufus* (reddish, red) and *manus* (hand), referring to the distinctly reddish pedipalpal hand (chela).

**Diagnosis.** This species most closely resembles *Allochthonius liaoningensis* Hu and Zhang, 2012 in the following characteristics: carapace with 24 setae, number of marginal teeth on the chelal finger (20 in *A. liaoningensis*, 17–20 in fixed finger, 15–18 in movable finger of *A*. *rufimanus* **sp. nov.**). Both species differ by the following characteristics: number of setae on the cheliceral palm (five in *A*. *rufimanus* **sp. nov.** *vs* six in *A. liaoningensis*), L/W ratio of leg I tarsus (7.77–8.94x in *A*. *rufimanus* **sp. nov.** *vs* 9.17–10x in *A. liaoningensis*), and L/W ratio of leg IV tarsus (7.26–8.51x in *A*. *rufimanus* **sp. nov.** *vs* 9.17–10.4x in *A. liaoningensis*).

**Description.**

**Holotype, male, adult (Figs. 13A–13B)**

Colour. Body light brown; pedipalps weakly sclerotized; tip of pedipalp red.

**Figure 14:**
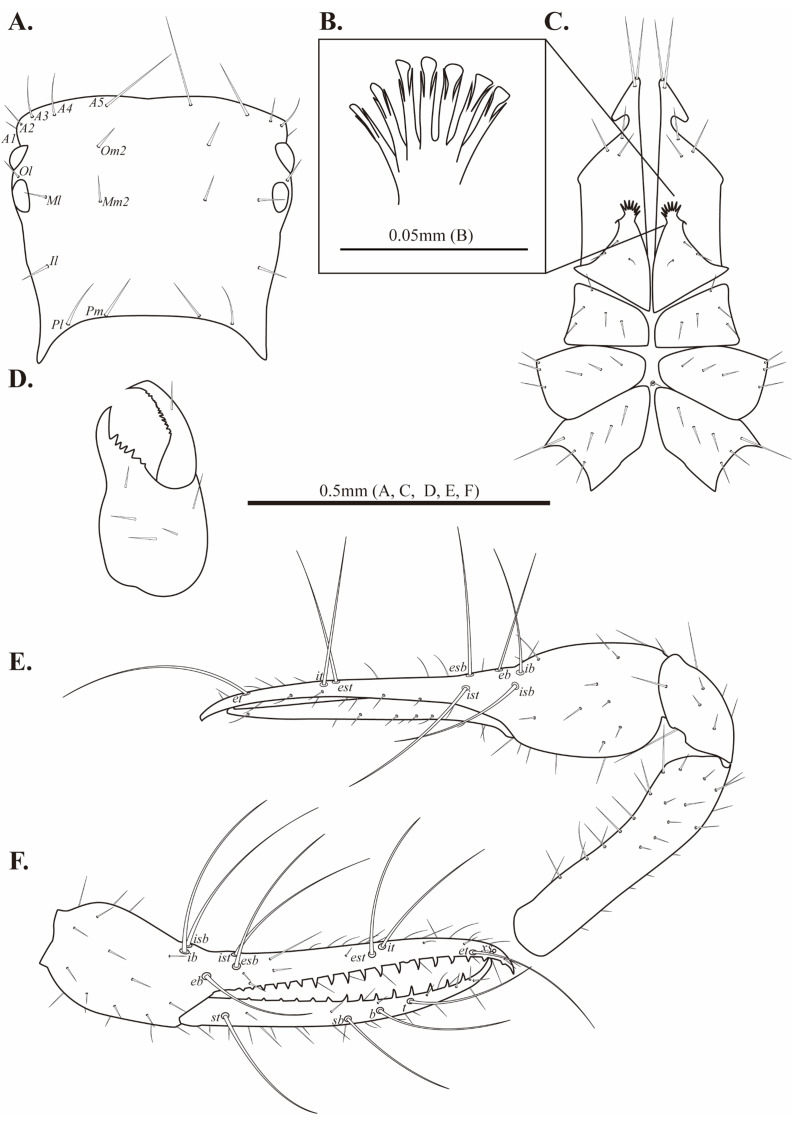
Drawings of *Allochthonius rufimanus* sp. nov. (A) Carapace, dorsal view; (B) coxal spines; (C) coxa; (D) right chelicera, dorsal view; (E) right pedipalp, dorsal view; (F) right chela, lateral view.

**Figure 15:**
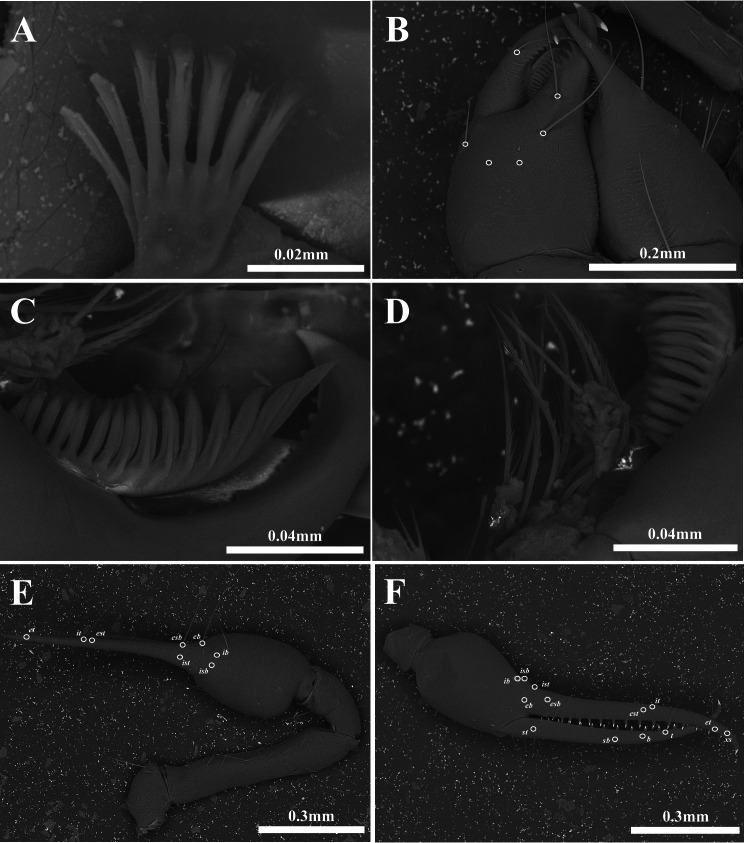
Scanning electron images of *Allochthonius rufimanus* sp. nov. (A) Coxal spines; (B) left chelicera, dorsal view; (C) serrula exterior; (D) rallum; (E) right pedipalp, dorsal view; (F) right chela, lateral view.

Cephalothorax (Figs. 14A–14C, 15A). Carapace 0.98 times longer than broad; rectangular; four distinct eyes; epistome absent; 24 setae on the carapace; five pairs of setae on the anterior margin (*A1*, *A2*, *A3*, *A4*, *A5*), a pair of setae on the lateral margin (*Ol*), four setae pairs on the medial margin (*Om2*, *Ml*, *Mm2*, *Il*), two pairs of setae on the posterior margin (*Pl*, *Pm*). Two acuminate setae on the manducatory process, maxilla with three setae; coxal chaetotaxy 3: 5: 6: 6; coxal spines with seven blades; bisetose intercoxal tubercle present between coxa III and IV.

Chelicera (Figs. 14D, 15B–15D). Palm smooth; five setae on the palm, one seta on the movable finger; six large marginal teeth on the fixed finger, 16 small marginal teeth on the movable finger; rallum with eight blades; serrula exterior with 15 blades.

Pedipalp (Figs. 14B–14F, 15E–15F). Trochanter 1.38 times, femur 4.91 times, patella 1.92 times, chela 4.71 times, hand 0.18 times longer than broad, movable finger 0.9 times longer than hand. Fixed finger with eight trichobothria, movable finger with four trichobothria; *isb* and *ib* basally positioned on the dorsum of the fixed finger; *eb* positioned on the lateral of the fixed finger; *ist* and *esb* grouped together, about one areolar apart; *it* and *est* positioned at the middle of the fixed finger; *et* positioned next to *xs*; *xs* terminally located on the fixed finger; *sb*, *b*, and *t* separately positioned from *st*; *st* basally positioned on the movable finger. Fixed finger with 21, movable finger with 17 marginal teeth.

Legs. Leg I: trochanter 1.63 times, femur 3.86 times, patella 3 times, tibia 3 times, tarsus 8 times longer than broad. Leg IV: trochanter 1.45 times, femur+patella 2.53 times, tibia 3.67 times, metatarsus 3 times, tarsus 10 times longer than broad; arolium undivided; pseudotactile setae present basally on leg IV metatarsus and tarsus.

Abdomen. Pleural membrane granulate; tergites undivided; tergal chaetotaxy 4: 4: 6: 6: 6: 8: 8: 10: 8: 6: 2: 0; sternite III–V divided; sternal chaetotaxy 8: 20: 20: 16: 14: 14: 14: 12: 6: 2: 2.

Dimensions (in mm). Body length 1.47. Pedipalp 0.18/0.13, femur 0.54/0.11, patella 0.23/0.12, chela (with pedicel) 0.80/0.17, movable finger 0.48, hand 0.3/0.17. Chelicera: total 0.36/0.18, movable finger 0.18, hand 0.2. Carapace 0.41/0.42; anterior eye 0.04, posterior eye 0.03. Leg I: trochanter 0.13/0.08, femur 0.27/0.07, patella 0.18/0.06, tibia 0.15/0.05, tarsus 0.32/0.04. Leg IV: trochanter 0.16/0.11, femur+patella 0.43/0.17, tibia 0.33/0.09, metatarsus 0.15/0.05, tarsus 0.3/0.03.

**Paratype, male, adult (*n* = 12)**

Cephalothorax. Carapace 0.92–1 time longer than broad; with 24 setae; 10 setae on the anterior margin, and four setae on the posterior margin. Coxal chaetotaxy 3–5: 5–7: 4–6: 6–7; coxa I with eight blades.

Pedipalp. Trochanter 1.4–1.41 times, femur 4.62–4.86 times, patella 1.79 times, chela 4.57–4.62 times, hand 1.74–1.78 times longer than broad, movable finger 1.56–1.66 times longer than the hand. Fixed finger with 18–19, movable finger with 16–19 marginal teeth.

Legs. Leg I: trochanter 1.31–1.47 times, femur 4.12–4.28 times, patella 2.67–3.12 times, tibia 3.04–3.27 times, tarsus 7.9–8.94 times longer than broad. Leg IV: trochanter 1.4 times, femur+patella 2.49–2.74 times, tibia 3.85–3.89 times, metatarsus 2.61–2.8 times, tarsus 7.58–7.78 times longer than broad.

Abdomen. Tergal chaetotaxy 4: 4: 6: 6: 6: 7–8: 8–10: 10: 6–7: 6: 0–2: 0; sternal chaetotaxy 8–9: 27–32: 16–20: 16–18: 14–15: 14: 12–15: 10–11: 6–7: 2–4: 2.

Dimensions (in mm). Body length 1.43–1.47. Pedipalp: trochanter 0.17–0.18/0.12–0.13, femur 0.53–0.54/0.11, patella 0.21–0.22/0.11–0.12, chela (with pedicel) 0.79–0.81/0.17–0.18, movable finger 0.49–0.5, hand 0.3–0.31/0.17–0.18. Chelicera: total 0.35–0.37/0.18–0.19, movable finger 0.19–0.2, hand 0.16–0.17/0.18–0.19. Carapace 0.38–0.40/0.40–0.42; anterior eye 0.04; posterior eye 0.04. Leg I: trochanter 0.12–0.14/0.08–0.1, femur 0.24–0.28/0.06–0.07, patella 0.16–0.19/0.06, tibia 0.14–0.16/0.05, tarsus 0.31–0.32/0.04. Leg IV: trochanter 0.17/0.12, femur+patella 0.41–0.46/0.16–0.17, tibia 0.31–0.34/0.08–0.09, metatarsus 0.14–0.15/0.05–0.06, tarsus 0.3/0.04.

**Paratypes, female, adult (*n* = 9) (Figs. 13C–13D)**

Cephalothorax. Carapace 0.79–0.91 times longer than broad; with 24 setae; 10 setae on the anterior margin, and four setae on the posterior margin. Coxal chaetotaxy 3–4: 5–6: 5–6: 5–7; coxal spines with eight blades.

Pedipalp. Trochanter 1.33–1.37 times, femur 4.28–4.48 times, patella 1.7–1.93 times, chela 3.93–4.01 times, hand 1.42–1.61 times longer than broad, movable finger 1.44–1.83 times longer than hand. Fixed finger with 17–20, movable finger with 15–18 marginal teeth.

Legs. Leg I: trochanter 1.13–1.18 times, femur 4.49–4.85 times, patella 2.95–3.39 times, tibia 3.54–3.81 times, tarsus 7.77–8.92 times longer than broad. Leg IV: trochanter 1.37–1.39 times, femur+patella 3.28–3.4 times, tibia 3.79–4.04 times, metatarsus 2.66–2.88 times, tarsus 7.26–8.51 times longer than broad.

Abdomen. Tergites undivided; tergal chaetotaxy 4: 4: 6: 6–7: 7: 8–9: 9: 9–10: 8: 6: 0: 0; sternites III–IV divided; sternal chaetotaxy 8–10: 20–22: 12–20: 14–16: 14: 12–15: 12–16: 12: 6: 2: 2.

Dimensions (in mm). Body length 1.59–1.67. Pedipalp: trochanter 0.19–0.21/0.14–0.16, femur 0.56–0.62/0.13–0.14, patella 0.24–0.25/0.12–0.15, chela (with pedicel) 0.94–0.96/0.24, movable finger 0.56–0.62, hand 0.34–0.38/0.24. Chelicera: total 0.43–0.44/0.22, movable finger 0.23–0.24, hand 0.2/0.22. Carapace 0.38–0.44/0.48; anterior eye 0.04–0.05; posterior eye 0.04–0.05. Leg I: trochanter 0.12–0.13/0.1–0.11, femur 0.32–0.35/0.07, patella 0.18–0.21/0.06, tibia 0.18/0.05, tarsus 0.33–0.34/0.04. Leg IV: trochanter 0.19/0.14, femur+patella 0.50–0.51/0.15–0.16, tibia 0.32–0.36/0.09, metatarsus 0.16/0.06, tarsus 0.28–0.33/0.04.

**Sequence data.** GenBank Accession No. PX533173. This species differs from other Korean species by 17% (*A. jungsuni* **sp. nov.**) to 24.3% (*A. maximus* **sp. nov.**) pairwise divergence in the CO1 data respectively.

### Interspecific pairwise genetic distances

Recent studies indicate that about 7% of genetic differences in the CO1 gene could delineate pseudotyrannochthoniids (Jeong et al., 2025; Harms et al., 2025). We also adapted this value since those studies were also focused on the same family, Pseudotyrannochthoniidae. Our interspecific pairwise genetic distance supports the species hypothesis derived from morphology and implies the presence of at least five species of *Allochthonius* in the Republic of Korea (Table 1). Genetic distances range between 13.2% to 24.3%. The pairwise genetic distance between *A. buanensis* and *A. jungsuni* **sp. nov.** is 13.2% and the genetic distance between *A. rufimanus* **sp. nov.** and *A. maximus* **sp. nov.** is 24.3%.

## Discussion

### Taxonomy of *Allochthonius* in South Korea

Pseudoscorpiones has been understudied in Korea, and the taxonomy is still in a state of flux, despite some recent advances (You et al., 2022; Jeong et al., 2025). Although *Allochthonius* is the most commonly collected pseudoscorpion genus in Korea and also the most diverse genus in the family Pseudotyrannochthoniidae, only three species have been reported from the Korean Peninsula to date. The first study was conducted by Morikawa (1970), who discovered *A. coreanus* and treated it as a subspecies of *A. opticus*. Lee (1982) subsequently described *A. buanensis*, but the diagnosis relied on a few characters such as the number of carapacal setae. Bang (1984) later elevated *A. coreanus* to full species rank, and Lee & Seo (1995) reported *A. opticus*, a species originally described from Japan, from Korea. However, as noted above, all Korean records were based on limited examinations and the original type materials are no longer available Therefore, we conducted an integrative taxonomic revision based on newly collected specimens from type localities.

The occurrence of *Allochthonius opticus* in Korea has long been uncertain. Originally described from Japan by Ellingsen (1907) and later subdivided into several subspecies by Morikawa (1960), *A. opticus* was reported from Korea only by Lee & Seo (1995). Their specimens were not assigned to either of the Japanese subspecies recognized by Morikawa (1956) –*Allochthonius opticus opticus* (Elligsen, 1907) and *A. opticus troglophilus* (Morikawa, 1956), the latter collected from Taniai-mura, Gifu Prefecture –lacked detailed comparison and are no longer available for study. Comparison of their descriptions with *A. opticus* **s. str.** reveals differences in setal counts and appendage size (Table 2), suggesting that the Korean material is unlikely to be conspecific. Given the absence of verifiable Korean material, the taxonomic identity of the reported specimens cannot be confirmed. *Allochthonius opticus* is therefore excluded from the Korean fauna in the present revision.

**Table 2:** Morphological comparison between Korean *Allochthonius opticus* and two morphological forms of *A. opticus* s. str.

	Korean *A. opticus*	Young adult form of *A. opticus*** s. str.**	Old adult form of *A. opticus*** s. str.**
Number of carapacal setae	10-4: 28	10-4: 26	10-4: 28
Number of setae on tergite I	Four	Six	Four
Length of chela	<1.3 mm	<1.3 mm	>1.4 mm
Length of pedipalpal femur	<0.9 mm	<0.9 mm	>0.9 mm

### Taxonomic status of Japanese *Allochthonius* subspecies

Almost all molecular studies indicate that basal lineages of pseudoscorpions, such as members of the suborder Heterosphyronida, exhibit narrow-range endemism, which can promote speciation and diversification, specifically in isolated terrains such as mountain ranges, forest fragments, and karst systems (Edward & Harvey, 2008; Harms, Roberts & Harvey, 2019; Hlebec et al., 2023; Harms et al., 2025). Recent advances in pseudoscorpion taxonomy further suggest that subtle morphological differences are indicative of species status and replace rather broad historical concepts of morphologically variable species (Hlebec et al., 2023; Hlebec et al., 2024; Muster et al., 2024). Following a recent example (You et al., 2022) in which all subspecies of the Asian genus *Spelaeochthonius* were elevated to full species rank, we likewise update historical species concepts for *Allochthonius* and elevate the subspecies described by Morikawa (1954); Morikawa (1956); Morikawa (1960) to full species level.

Although Morikawa’s type specimens are currently inaccessible, the original taxonomic descriptions are fairly complete, and the subspecies can be distinguished by discrete diagnostic morphological characters that clearly justify species-level recognition today (Morikawa, 1960). Originally, *Allochthonius ishikawai* (Morikawa, 1954) comprised six subspecies: *A. i. deciclavatus* Morikawa, 1956; *A. i. ishikawai* Morikawa, 1954; *A. i. kyushuensis* Morikawa, 1960; *A. i. shiragatakiensis* Morikawa, 1954; *A. i. uenoi* Morikawa, 1956; and *A. i. uyamadensis* (Morikawa, 1954). We elevate all these subspecies to species-level: *Allochthonius deciclavatus* **stat. nov.**, *Allochthonius kyushuensis* **stat. nov.**, *Allochthonius shiragatakiensis* **stat. nov.**, *Allochthonius uenoi* **stat. nov.**, and *Allochthonius uyamadensis* **stat. nov.** Additionally, two subspecies have been recognized under *Allochthonius opticus* Ellingsen, 1907: *A. o. opticus* Ellingsen, 1907 and *A. o. troglophilus* Morikawa, 1956. We elevate *A. o. troglophilus* to species level as *Allochthonius troglophilus* **stat. nov.** An identification key for the taxa formerly regarded as subspecies of *A. ishikawai* and *A. opticus* is presented in the ‘Identification Key’. With this step, we have simplified the taxonomy of Pseudotyrannochthoniidae in Asia and are able to provide biologically and taxonomically more meaningful species concepts. In summary, *Allochthonius* now includes 43 species in Japan (15 species), China (14 species), Taiwan (seven species), Russia (one species), and Korea (five species). It is the most diverse genus of the Pseudotyrannochthoniidae with potentially many more species to be discovered in the future.

### Cryptic diversity of *Allochthonius* in Korea

The present study provides a taxonomic baseline for more work on *Allochthonius* in Korea and adds three new species to the country record (Morikawa, 1970; Lee, 1982; Lee & Seo, 1995); this study). This is still a low number compared to those in neighboring countries, and we suggest that more species will be described from the Korean Peninsula in the future. *Allochthonius* is a genus that can be found in both epigean and subterranean habitats (Morikawa, 1960; Viana & Ferreira, 2021; Gao, Hou & Zhang, 2023). In neighboring countries, such as China and Japan, several subterranean species are included (Morikawa, 1954; Morikawa, 1956; Viana & Ferreira, 2021)(Gao Hou & Zhang, 2024; Li, 2023). However, on the Korean Peninsula, no subterranean *Allochthonius* has yet been discovered. Considering that the Korean Peninsula possesses more than a thousand caves, including both limestone karsts and lava tubes (Woo et al., 2015; Woo & Kim, 2018), it is likely that *Allochthonius* could be distributed underground in Korea. Moreover, even among epigean species, a barcoding study suggested cryptic species within *Allochthonius buanensis* a species complex (Ohira et al., 2018). In summary, considering the landscape of the Korean Peninsula and related studies, it is likely that a high diversity of *Allochthonius* is inherent to this region. Therefore, extensive field surveys and molecular work are necessary to unveil the diversity of this fauna.

### Identification key

**Identification key differentiating former subspecies of *Allochthonius ishikawai*:**

**Table:** 

1. Pedipalpal femur 5.1–5.4 times longer than broad …*A. deciclavatus*** stat. nov.** (Matsubarano-ana cave, Yamaguchi Prefecture, Japan)
- Pedipalpal femur 5.8–7.5 times longer than broad …2
2. Five pairs of setae on the anterior margin of carapace (A0–A5) …*A. kyushuensis*** stat. nov.** (Goya daiichi-do cave, Fukuoka Prefecture, Japan)
- Four pairs of setae on the anterior margin of carapace (A0–A4) …3
3. 18 setae on the carapace …*A. uyamadensis*** stat. nov.** (Uyama-do cave, Okayama Prefecture, Japan)
- More than 19 setae on the carapace …4
4. 24 setae on the carapace; carapace more than 0.6 mm …*A. uenoi*** stat. nov.** (Komakado-kaza-ana cave, Shizuoka Prefecture, Japan)
- 20 setae on the carapace; carapace less than 0.6 mm …5
5. 13–16 marginal teeth on both chelal finger; fingers not curved …*A. ishikawai* (Ryuga-do cave, Kochi Prefecture, Japan)
- 9–11 marginal teeth on both chelal finger; fingers strongly curved …*A. shiragatakiensis*** stat. nov.** (Shiragataki-do cave, Ehime Prefecture, Japan)

**Identification key differentiating former subspecies of *Allochthonius opticus***

**Table:** 

1. Pedipalpal femur 4.4–5.5 times longer than broad …*A. opticus* (Okayama, Okayama Prefecture, Japan)
- Pedipalpal femur 5.5–6.1 times longer than broad …*A. troglophilus*** stat. nov.** (Kugo cave, Gifu Prefecture, Japan)

## Conclusions

The genus *Allochthonius* is widely distributed in East Asia; however, its diversity in the Republic of Korea has remained poorly studied since the 1990s. In this study, we revise Korean *Allochthonius* using an integrative taxonomic framework and describe three new species, *A. jungsuni* **sp. nov.**, *A. maximus* **sp. nov.**, and *A. rufimanus* **sp. nov.**, each supported by diagnostic morphological characters. Interspecific pairwise genetic distances further corroborate the presence of at least five distinct *Allochthonius* species in Korea.

Our comparative morphological analyses and review of previous taxonomic literature support the elevation of several subspecies to species rank, including *A. deciclavatus* **stat. nov.**, *A. kyushuensis* **stat. nov.**, *A. shiragatakiensis* **stat. nov.**, *A. uenoi* **stat. nov.**, *A. uyamadensis* **stat. nov.**, and *A. troglophilus* **stat. nov.** Conversely, *A. opticus* is excluded from the Korean fauna based on clear morphological discrepancies with *A. opticus **sensu** stricto*.

Although relatively few species of *Allochthonius* are currently recognized from Korea, the peninsula’s complex geography and molecular evidence suggest the presence of additional, potentially cryptic diversity. Continued integrative taxonomic studies incorporating extensive sampling, detailed morphology, and molecular data will be essential for a comprehensive understanding of Korean pseudoscorpion diversity.

## Supplemental Information

10.7717/peerj.21332/supp-1 Supplemental Information 1 Sequences dataset

## Additional Information and Declarations

### Data Availability

The following information was supplied regarding data availability: The sequences used in this study are available at GenBank: PX533168, PX921017, and PX533173.

### New Species Registration

The following information was supplied regarding the registration of a newly described species: Publication LSID: urn:lsid:zoobank.org:pub:A53E1EB1-DE99-45D8-B92B-E538649EEB13 Allochthonius jungsuni: urn:lsid:zoobank.org:act:EA291957-FD99-4772-8DD1-C7E566C242D8 Allochthonius maximus: urn:lsid:zoobank.org:act:3C5116C3-D9BB-4B2B-90F1-777F0DC48554 Allochthonius rufimanus: urn:lsid:zoobank.org:act:9C6953B6-1649-4129-A09F-D5501270B933.
